# DNA methylation safeguards the generation of hematopoietic stem and progenitor cells by repression of Notch signaling

**DOI:** 10.1242/dev.200390

**Published:** 2022-05-25

**Authors:** Yan Li, Chao Tang, Fan Liu, Caiying Zhu, Feng Liu, Ping Zhu, Lu Wang

**Affiliations:** 1 State Key Laboratory of Membrane Biology, Institute of Zoology, Institute for Stem Cell and Regeneration, Chinese Academy of Sciences, University of Chinese Academy of Sciences, Beijing, 100101, China; 2 State Key Laboratory of Experimental Hematology, National Clinical Research Center for Blood Diseases, Haihe Laboratory of Cell Ecosystem, Institute of Hematology and Blood Diseases Hospital, Chinese Academy of Medical Sciences and Peking Union Medical College, Tianjin, 300020, China

**Keywords:** DNA methylation, Dnmt1, Notch, Hematopoietic stem and progenitor cell, Zebrafish

## Abstract

The earliest hematopoietic stem and progenitor cells (HSPCs) are generated from the ventral wall of the dorsal aorta, through endothelial-to-hematopoietic transition during vertebrate embryogenesis. Notch signaling is crucial for HSPC generation across vertebrates; however, the precise control of Notch during this process remains unclear. In the present study, we used multi-omics approaches together with functional assays to assess global DNA methylome dynamics during the endothelial cells to HSPCs transition in zebrafish, and determined that DNA methyltransferase 1 (Dnmt1) is essential for HSPC generation via repression of Notch signaling. Depletion of *dnmt1* resulted in decreased DNA methylation levels and impaired HSPC production. Mechanistically, we found that loss of *dnmt1* induced hypomethylation of Notch genes and consequently elevated Notch activity in hemogenic endothelial cells, thereby repressing the generation of HSPCs. This finding deepens our understanding of HSPC specification *in vivo*, which will provide helpful insights for designing new strategies for HSPC generation *in vitro*.

## Abstract (precis)

**Summary:** Multi-omics approaches and functional assays reveal global DNA methylome dynamics and an indispensable role of DNA methyltransferase 1 in hematopoietic stem/progenitor cell generation through repression of Notch signaling.

## INTRODUCTION

In vertebrates, hematopoietic stem and progenitor cells (HSPCs) are generated from a subset of endothelial cells (ECs), termed hemogenic endothelium, through endothelial-to-hematopoietic transition (EHT) during embryogenesis (Bertrand et al., 2010; Boisset et al., 2010; Kissa and Herbomel, 2010). During this transition, hemogenic endothelial cells (HECs) change cell morphology and tune down the arterial program to acquire hematopoietic identity (Gama-Norton et al., 2015; Lizama et al., 2015). Identification of possible activators of the hemogenic program and silencers of arterial identity is crucial for understanding the mechanism underlying HSPC generation, which is of great clinical importance for *in vitro* blood production in regenerative medicine. However, our comprehensive understanding of the global transcriptional regulation of the EHT program in vertebrate models remains incomplete.

Multiple factors and signaling pathways have been identified as essential regulators for EHT, including transcription factors Runx1 (Bonkhofer et al., 2019; Chen et al., 2009), Sox17 (Lizama et al., 2015) and Notch signaling (Gama-Norton et al., 2015; Lizama et al., 2015; Zhang et al., 2015). Among these, Notch signaling is at the core of the complex regulatory mechanisms in controlling HSPC development (Bigas et al., 2013; Gama-Norton et al., 2015; Lomeli and Castillo-Castellanos, 2020). Notch signaling regulates HEC specification or HSPC generation through downstream factors, including *runx1* (Bresciani et al., 2014), *cbfb* (Bresciani et al., 2014), *cdca7a* (Guiu et al., 2014) and *Gata2* (Robert-Moreno et al., 2005), in a cell-autonomous or non-cell-autonomous manner (Clements et al., 2011; Kim et al., 2014; Kobayashi et al., 2014; Lomeli and Castillo-Castellanos, 2020). Previous studies have indicated that Notch signaling is not continuously required during HSPC generation, and that it should be downregulated to facilitate HSPC emergence immediately after HEC specification (Gama-Norton et al., 2015; Lizama et al., 2015; Zhang et al., 2017; Zhang et al., 2015). One mechanism for Notch signaling downregulation has been reported in zebrafish, in which G protein-coupled receptor 183 (Gpr183) recruits β-arrestin1 and Nedd4 to degrade Notch1 through the proteasome pathway in HECs (Zhang et al., 2015). Meanwhile, Blos2 (a subunit of biogenesis of lysosome-related organelles complex-1, BLOC-1), which plays a role in endocytic trafficking, can negatively regulate Notch signaling in zebrafish and mouse embryos (Zhou et al., 2016). Loss of Blos2 leads to the impaired degradation of Notch1, thereby causing excessive activation of Notch activity and HSPC defects (Zhou et al., 2016). Regulation of mRNA stability is also reported to be involved in Notch turnover. *notch1a* mRNA with N6-methyladenosine (m^6^A) modification could be recognized by Ythdf2, which is a specific reader to mediate m^6^A-modified mRNA decay, thereby downregulating Notch signaling during HSPC generation (Lv et al., 2018; Zhang et al., 2017). Although the upstream regulatory mechanism of Notch signaling after the initiation of EHT has been extensively studied, the precise regulation of Notch activity, especially at the epigenetic level, is still largely unknown.

DNA methylation plays important roles in gene regulation, genomic imprinting, X-chromosome silencing and embryonic development (Bird, 2002; Cedar and Bergman, 2012; Luo et al., 2018; Wang et al., 2017). DNA methylation is established by *de novo* methyltransferases, DNMT3A and DNMT3B, and is maintained by DNA methyltransferase 1 (DNMT1) (Smith and Meissner, 2013). Accumulating evidence suggests that the regulation of DNA methylation by DNMTs is crucial for adult hematopoiesis, including HSPC self-renewal, expansion and lineage differentiation (Broske et al., 2009; Challen et al., 2011, 2014; Izzo et al., 2020; Trowbridge et al., 2009). In contrast, the regulatory role of DNA methylation in the generation of HSPCs from HECs is still underexplored. Two previous studies in zebrafish reported that *de novo* methyltransferase 3bb.1 (Dnmt3bb.1) promotes the maintenance of HSPC fate via methylation of the key transcription factor *cmyb* (also known as *myb*), and that Dnmt1 regulates HSPC formation by altering *cebpa* expression (Gore et al., 2016; Liu et al., 2015), highlighting the important role of DNA methyltransferases in HSPC emergence during embryonic development. However, the mechanism by which genetic and epigenetic regulatory modes are established at a genome-wide scale to spatio-temporally regulate cell fate transition during HSPC generation remains elusive.

In the present study, we investigated the role of DNA methylation in HSPC generation in zebrafish. We generated a comprehensive DNA methylome landscape during normal embryogenesis and performed functional assays to determine how Notch signaling is precisely regulated at the epigenetic level. The findings of the present study illuminate the role of DNA methylation in modulation of Notch signaling during HSPC generation in zebrafish embryos.

## RESULTS

### Dynamic DNA methylation during HSPC generation

**Fig. 1.**
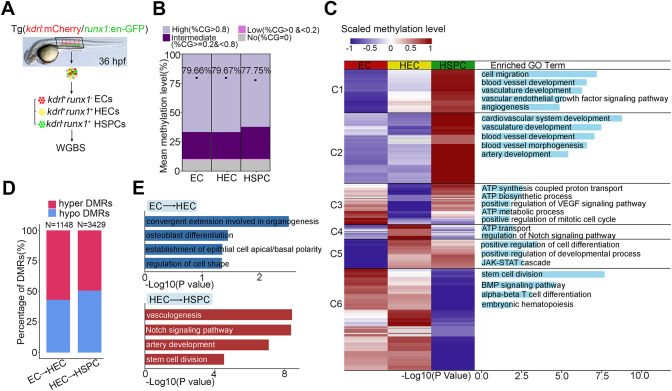
**Dynamic DNA methylation during HSPC generation.** (A) Flowchart of sorting and whole genome bisulfite sequencing. EC, endothelial cell; HEC, hemogenic endothelial cell; HSPC, hematopoietic stem and progenitor cell; WGBS, whole genome bisulfite sequencing. (B) Global DNA methylation levels in EC, HEC, HSPC and fraction of CpGs displaying high (>0.8), intermediate (≥0.2 and <0.8), low (>0 and <0.2) and no (=0) 5mC. *n*=3 replicates. (C) Heatmap displays differentially methylated regions (DMRs) in EC, HEC and HSPC. Right column shows functional enrichment of DMR-related genes by GREAT analysis. The *x*-axis represents the negative log of the *P*-values of the enrichment of the corresponding GO terms. C1-C6, Clusters 1-6. (D) The percentage of hyper-DMRs and hypo-DMRs between each two consecutive stages: EC versus HEC and HEC versus HSPC. (E) GO enrichment of DMRs by GREAT analysis between each two consecutive stages: EC versus HEC and HEC versus HSPC. The *x*-axis represents the negative log of the *P*-values of the enrichment of the corresponding GO terms.

To determine the DNA methylation landscape dynamics during HSPC generation in zebrafish, we performed whole genome bisulfite sequencing (WGBS) to obtain the methylomes of ECs, HECs and HSPCs sorted from the aorta-gonad-mesonephros (AGM) region of the transgenic line *kdrl*:mCherry/*runx1*:en-GFP at 36 h post fertilization (hpf), when EHT occurred frequently (Fig. 1A). ECs, HECs and HSPCs were globally and highly methylated, with average 5mC levels of 79.66%, 79.67% and 77.75%, respectively (Fig. 1B). We identified cell-type-specific differentially methylated regions (DMRs), in which most EC-specific and HEC-specific DMRs were hypomethylated, whereas a large proportion of HSPC-specific DMRs were hypermethylated (Fig. S1A). The distribution of specific DMRs in these three cell types was mainly mapped to introns and gene body regions (Fig. S1B). To characterize the comprehensive methylomes of EC, HEC and HSPC, we categorized DMRs that were obtained by pairwise comparison of each two adjacent cell types into six distinct clusters based on their methylation levels and used the Genomic Regions Enrichment of Annotations Tool (GREAT) to perform gene ontology (GO) enrichment analysis (Fig. 1C). DMRs in Clusters 1 and 2 showed specific hypermethylation in HSPCs and related genes were mainly enriched in vasculature development, indicating a potential DNA methylation mediated-inhibition of endothelial identity-associated genes during EHT. DMRs in Cluster 1 indicated a gradual DNA methylome formation of genes from ECs to HSPCs, such as cell migration, blood vessel development and angiogenesis-related genes (Fig. 1C). The enriched GO terms of the DMRs in Cluster 3, which were specifically hypomethylated in HECs, included ATP biosynthetic processes, which are involved in HSPC generation. In contrast to Cluster 3, DMRs in Cluster 4 were highly and specifically methylated in HECs, and GO enrichment analysis suggested enrichment in regulation of Notch signaling, indicating that the changes in methylation in regions near these genes were associated with the repression of Notch activity in HECs. Specific hypomethylated DMRs in ECs and HSPCs were grouped into Clusters 5 and 6, respectively. DMRs in Cluster 5 showed enrichment in terms of positive regulation of cell differentiation and JAK-STAT cascade, whereas stem cell division, T cell differentiation and embryonic hematopoiesis were enriched in Cluster 6 (Fig. 1C). Collectively, dynamic methylation indicated a potential role of DNA methylation during HSPC generation.

In detail, we focused on two consecutive stages, hematopoietic specification (EC versus HEC) and hematopoietic generation (HEC versus HSPC), to further describe the methylation changes during HSPC generation. We compared changes in the methylomes in these two stages, and identified 1148 and 3429 DMRs, respectively (Fig. 1D). GO analysis of DMRs between EC and HEC revealed enrichment in regulation of cell shape, whereas the enriched terms of HEC versus HSPC included vasculogenesis, Notch signaling, artery development and stem cell division, indicating the continuous role of DNA methylation in the regulation of HSPC generation (Fig. 1E). Taken together, these data reveal dynamic DNA methylation changes in specific genomic regions that are likely involved in the endothelial-to-hematopoietic fate transition during HSPC generation.

### Dnmt1 is required for HSPC generation cell autonomously

**Fig. 2.**
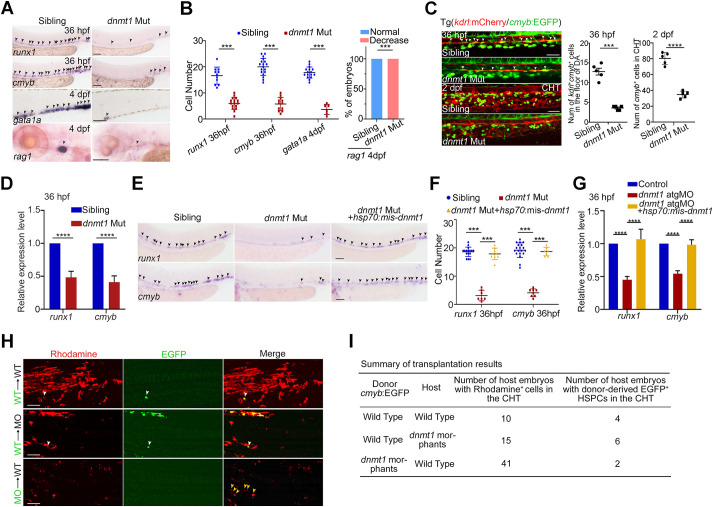
**DNA methylation is required for HSPC generation.** (A) Expression of HSPC markers *runx1* and *cmyb* (arrowheads) at 36 hpf, erythroid marker *gata1* (arrowheads) at 4 dpf and lymphoid marker *rag1* (arrowheads) at 4 dpf in siblings and *dnmt1* mutants by WISH. *n*≥3 replicates. (B) Quantification of WISH results in A. (C) Confocal imaging showing the number of *kdrl^+^*/*cmyb^+^* HECs in AGM at 36 hpf (white arrowheads), and *cmyb^+^* HSPCs in the caudal hematopoietic tissue (CHT) region at 2 dpf in siblings and *dnmt1* mutants (left panels, *n*≥3 replicates), with quantification (right panels). *n*=5 embryos. (D) qPCR analysis of *runx1* and *cmyb* expression in sibling and *dnmt1* mutant embryos at 36 hpf. *n*=3 replicates. (E) Expression of *runx1* and *cmyb* in sibling, *dnmt1* mutant and *dnmt1* mutant embryos injected with *hsp70*: mismatch-*dnmt1*-EGFP constructs. The arrowheads indicate the expression of HSPC markers *runx1* and *cmyb*. *n*≥3 replicates. (F) Quantification of WISH results in E. (G) qPCR analysis of *runx1* and *cmyb* expression in control, *dnmt1* morphants and embryos co-injected with *dnmt1*-atgMO and *hsp70*: mismatch-*dnmt1*-EGFP constructs. *n*=3 replicates. (H) Transplantation results showing HSPC reconstitution in the CHT region of recipient embryos at 36 hpf. Green, *cmyb*^+^ EGFP cells; red, Rhodamine. White arrowheads show EGFP^+^ HSPCs contributed by donor cells; yellow arrowheads show donor-derived EGFP^−^ hematopoietic cells in CHT region. (I) Summary of transplantation results in H. Data are mean±s.d. ***P<0.001, ****P<0.0001 (unpaired two-tailed Student's *t*-test). Scale bars: 100 μm (A,E); 50 μm (C,H).

Our data showed that among all DNA methyltransferase (DNMT) members in the zebrafish, *dnmt1* was co-expressed with *cmyb/runx1* (HSPC markers) in HSPCs at 36 hpf (Fig. S2A). We therefore chose *dnmt1* to perform knockout or knockdown assays to reduce methylation level as reported previously (Wang et al., 2017). In *dnmt1* mutant (*dnmt1*^s872^) embryos (Anderson et al., 2009), the expression of HSPC markers *runx1* and *cmyb* was almost entirely absent at 36 hpf (Fig. 2A,B), whereas the derivatives of HSPCs, erythroid and lymphoid cells (labeled by *gata1a* and *rag1*, respectively), were also impaired (Fig. 2A,B). Using Tg(*kdrl*:mCherry/*cmyb*:EGFP) embryos, we found that the number of HECs (*kdrl*^+^*cmyb*^+^) in the ventral wall of dorsal aorta was significantly reduced at 36 hpf, leading to a decrease of *cmyb*^+^ HSPCs in the caudal hematopoietic tissue (CHT) region at 2 days post fertilization (dpf) (Fig. 2C). Quantitative real-time PCR (qPCR) confirmed the decreased expression of *runx1* and *cmyb* at 36 hpf in *dnmt1* mutants compared with that in siblings (Fig. 2D). Furthermore, time-lapse imaging of EHT in *dnmt1*-deficient embryos showed that most ECs failed to transit into HSPCs, compared with the control with fate transition occurring (Movies S1 and S2). Next, we used the *dnmt1* translation-blocking morpholino (atgMO), which caused a decrease in Dnmt1 protein levels (Fig. S2B), to verify the aforementioned phenotypes. Whereas primitive hematopoiesis was relatively normal in *dnmt1* atgMO-injected embryos (Fig. S2C), the expression levels of *runx1* and *cmyb* at 36 hpf in the AGM region and *cmyb* at 3 dpf and 4 dpf in the CHT region were obviously reduced (Fig. S2D,E). qPCR analysis of *runx1* and *cmyb* (Fig. S2F) and immunoblotting analysis of Runx1 at 36 hpf (Fig. S2B) confirmed the whole mount *in situ* hybridization (WISH) results, similarly suggesting impaired HSPC emergence. The populations of *kdrl*^+^*cmyb*^+^ HECs in the AGM region at 36 hpf and *cmyb*^+^ HSPCs in the CHT region at 2 dpf were markedly decreased upon *dnmt1* deficiency (Fig. S2G). HSPC derivatives, such as erythrocytes (labeled by *ae1-globin*; *hbae1.1*), myeloid cells (labeled by *l-plastin*; *lcp1*), and lymphoid cells (labeled by *rag1*), were all impaired in *dnmt1* morphants (Fig. S2H,I). A splice-blocking MO (sMO) of *dnmt1* was also designed, and its knockdown efficiency was validated by immunoblotting (Fig. S3A). The unaffected primitive hematopoiesis and abnormal HSPC development in *dnmt1* sMO-injected embryos were consistent with those observed in *dnmt1* genetic mutants and atgMO-injected embryos (Fig. S3B-H). Collectively, these results indicate that Dnmt1 is required for HSPC development.

To evaluate the exact stage of DNA methylation functioning in definitive hematopoiesis, we performed time-course experiments with 5-Aza treatment, a specific inhibitor of DNA methyltransferases, from 24 hpf to 36 hpf, the time window during which HSPCs are generated through EHT (Bertrand et al., 2010; Kissa and Herbomel, 2010). The expression of *runx1* and *cmyb* was decreased in 5-Aza-treated embryos at 36 hpf, indicating that DNA methylation was required for HSPC generation (Fig. S4A,B). After validating the overexpression efficiency by live imaging and western blotting (Fig. S4C,D), we applied a stage-specific mRNA rescue approach, in which *dnmt1* was driven by the *hsp70* promoter, from 24 hpf onwards, and found that the HSPC defects in *dnmt1* mutant and morphants were efficiently rescued (Fig. 2E-G; Fig. S4E,F). Altogether, these results demonstrate that Dnmt1 is responsible for HSPC generation.

We next performed rescue experiments by co-injecting *dnmt1* atgMO and *dnmt1* mis-mRNA (with mutated atgMO-binding sites to avoid MO blocking) with or without the conserved methyltransferase domain (Fig. S4G). The decreased *runx1* expression in *dnmt1* morphants can be rescued by *dnmt1* mis-mRNA but not by the truncated mis-mRNA (Fig. S4H,I), suggesting that Dnmt1 regulation of HSPC development was dependent on its methyltransferase activity.

To further determine whether Dnmt1 is required cell autonomously for the generation of HSPCs, we performed cell transplantation at blastula stage. Rhodamine-labeled lateral mesodermal cells from Tg(*cmyb*:EGFP) embryos were transplanted into the corresponding regions of non-transgenic recipient embryos. The results showed that 4/10 wild-type recipients and 6/15 *dnmt1* morphant recipients had a few GFP^+^ HSPCs derived from wild-type donor cells, whereas only few wild-type recipients (2/41) had GFP^+^ HSPCs from donor cells of *dnmt1* morphants (Fig. 2H,I), suggesting that blastula cells lacking *dnmt1* hardly gave rise to *cmyb*^+^ HSPCs in normal recipients. Taken together, Dnmt1 is required for HSPC generation cell autonomously.

### DNA methylation controls HSPC generation through repressing endothelial identity

**Fig. 3.**
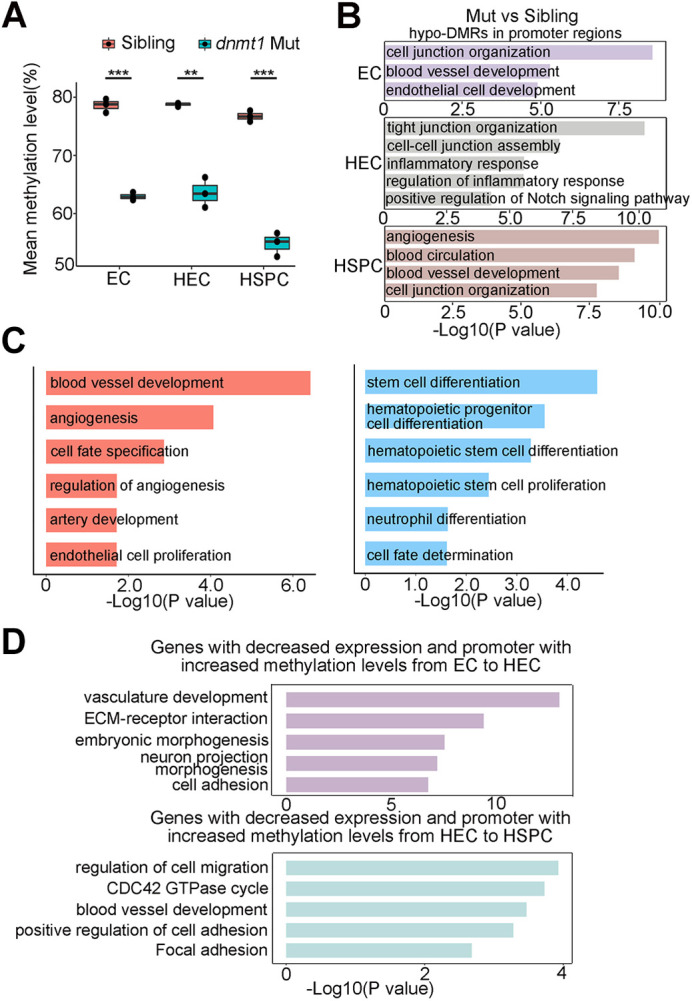
**Loss of Dnmt1 deregulates endothelial programs.** (A) Mean methylation level in EC, HEC and HSPC in siblings and *dnmt1* mutants shows decreased methylation level upon the depletion of *dnmt1*. Data are mean±s.d. *n*=3 replicates. **P<0.01, ***P<0.001 (unpaired two-tailed Student's *t*-test). The boxes show the interquartile range (IQR) around median (middle line). Whiskers correspond to 1.5x IQR. Dots indicate replicates=3. (B) GO analysis of genes with hypomethylated DMRs in promoter regions in *dnmt1* mutants, compared with siblings, in EC, HEC and HSPC. (C) GO enrichment of genes with differential expression showing the enrichment of upregulated (left panel) and downregulated (right panel) signaling pathways in *dnmt1* mutants. (D) GO analysis of the genes inactivated during HSPC generation, while the methylation levels of their promoters increased. The *x*-axis represents the negative log of the *P*-values of the enrichment of the corresponding GO terms (B-D).

To investigate the molecular mechanisms by which DNA methylation affects HSPC generation, we performed WGBS and RNA-sequencing (RNA-seq) of ECs, HECs and HSPCs sorted from siblings and *dnmt1* mutants at 36 hpf. Deletion of *dnmt1* caused a significant decrease of global DNA methylation level in ECs, HECs and HSPCs (from 79.66% to 63.62% in ECs, from 79.67% to 63.92% in HECs and from 77.75% to 55.07% in HSPCs) (Fig. 3A). Consistently, analysis of DMRs revealed that, compared with their siblings, *dnmt1* mutants had more hypomethylated DMRs (hypo-DMRs) than hypermethylated DMRs (hyper-DMRs) in three different cell types (Fig. S5A). The distribution of DMRs in these three cell types was mainly mapped to gene body and introns regions (Fig. S5B). We identified hypo-DMRs at the promoter region in ECs, HECs and HSPCs, and then performed GO analysis of these genes. Genes with hypo-DMRs in ECs were enriched in terms related to cell junction organization, blood vessel development and endothelial cell development. Hypo-DMRs in HECs were enriched in terms related to cell junction organization, inflammatory response and positive regulation of Notch signaling, which are all essential for EHT. Meanwhile, the enriched GO terms for hypo-DMRs in HSPCs included angiogenesis and blood vessel development (Fig. 3B). Together, these results suggest a potential role of Dnmt1-mediated DNA methylation in regulation of endothelial cell and blood vessel development during HSPC generation.

Next, we examined the expression level and found that 901 and 795 genes were upregulated and downregulated in *dnmt1* mutants*,* respectively (Fig. S5C). Intriguingly, genes with upregulated expression showed enrichment in blood vessel development, angiogenesis and artery development, whereas genes with decreased expression were enriched for terms associated with stem cell differentiation and hematopoietic progenitor cell differentiation (Fig. 3C). Besides, gene set enrichment analysis (GSEA) in HECs suggested that *dnmt1* deficiency resulted in enriched vasculogenesis and Notch signaling (Fig. S5D). Collectively, these results indicate that *dnmt1* deficiency led to dysregulated blood vessel development-related transcription, thereby disrupting HSPC generation.

To further interrogate whether Dnmt1-mediated methylation regulates gene expression during HSPC generation, we performed correlations analysis between methylation of promoter regions and corresponding gene transcription at hematopoietic specification stage (EC versus HEC) and hematopoietic generation stage (HEC versus HSPC) in sibling and *dnmt1* mutants, respectively. As DNA methylation usually acts as a repressive regulator of gene expression (Jones, 2012), we identified genes with negative correlation and further analyzed the genes with upregulated promoter methylation and decreased RNA expression in sibling samples, but not in *dnmt1* mutant. We identified 391 and 199 genes at specification and generation stages, respectively. GO analysis showed that these genes at specification and generation stages were both clearly enriched in the terms associated with vasculature development and blood vessel development, which indicated that Dnmt1-mediated methylation was involved in regulation of vessel development-related genes (Fig. 3D).

**Fig. 4.**
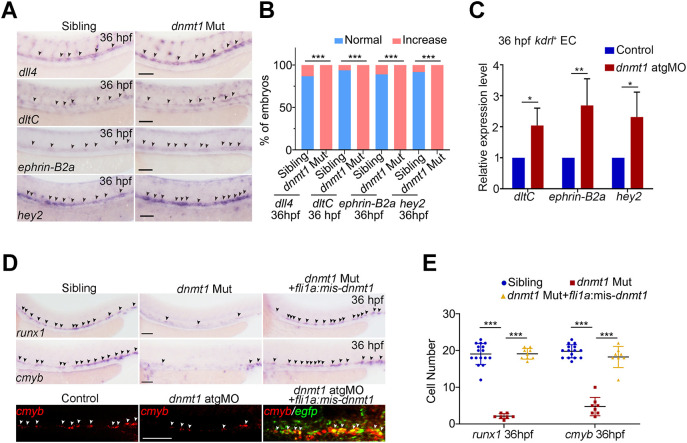
**Dnmt1-mediated methylation controls HSPC generation through blocking arterial endothelial identity.** (A) WISH analysis showing the expression of arterial endothelial genes *dll4*, *dltC*, *ephrin-B2a* and *hey2* in siblings and *dnmt1* mutants at 36 hpf. The arrowheads indicate the expression of corresponding arterial endothelial genes. *n*≥3 replicates. (B) Statistical analysis of the WISH in A. (C) qPCR analysis of arterial endothelial genes *dltC*, *ephrin-B2a* and *hey2* in *kdrl*^+^ ECs in siblings and *dnmt1* mutants. *n*=3 replicates. (D) WISH results showing expression of *runx1* and *cmyb* (black arrowheads) at 36 hpf in sibling, *dnmt1* mutant and *dnmt1* mutant embryos injected with *fli1a*: mismatch-*dnmt1*-EGFP constructs (upper panels) and FISH analysis of *cmyb* and *egfp* (white arrowheads) at 36 hpf in control, *dnmt1* morphants and embryos co-injected with *dnmt1* atgMO and *fli1a*:mismatch-*dnmt1*-EGFP constructs showing that endothelial-derived Dnmt1-EGFP overexpression rescued the population of *cmyb*^+^ cells (bottom panel). *n*=3 replicates. (E) Statistical analysis of the WISH data in D. Data are mean±s.d. *P<0.05, **P<0.01 ***P<0.001 (unpaired two-tailed Student's *t*-test). Scale bars: 100 µm (A,D upper panels); 50 µm (D bottom panels).

To further explore whether DNA methylation affects HSPC generation by regulating vascular genes, we first examined the expression of arterial endothelial markers *dll4*, *dltC*, *ephrin-B2a* and *hey2*. The expression of these genes was significantly increased in *dnmt1* knockout and knockdown embryos (Fig. 4A-C; Fig. S5E,F), suggesting that Dnmt1-mediated methylation inhibited arterial endothelial genes. Next, to determine whether the Dnmt1 deficiency-induced arterial genes were responsible for the HSPC defects, we performed endothelial overexpression of *dnmt1* by co-injecting constructs driven by *fli1a* promoter with *tol2* transposon (Fig. S5G). The results showed that *dnmt1* overexpression in ECs efficiently rescued the decrease of HSPCs in the AGM region in *dnmt1* mutants and morphants (Fig. 4D,E; Fig. S5G,H). In addition, double fluorescent *in situ* hybridization (FISH) of *cmyb* and *egfp* showed that endothelial-derived Dnmt1-EGFP overexpression rescued the population of *cmyb*^+^ cells (Fig. 4D). Taken together, our data suggest that DNA methylation actively promotes HSPC generation through the repression of arterial endothelial genes.

### Increased Notch signaling is involved in HSPC defects upon loss of DNA methylation

**Fig. 5.**
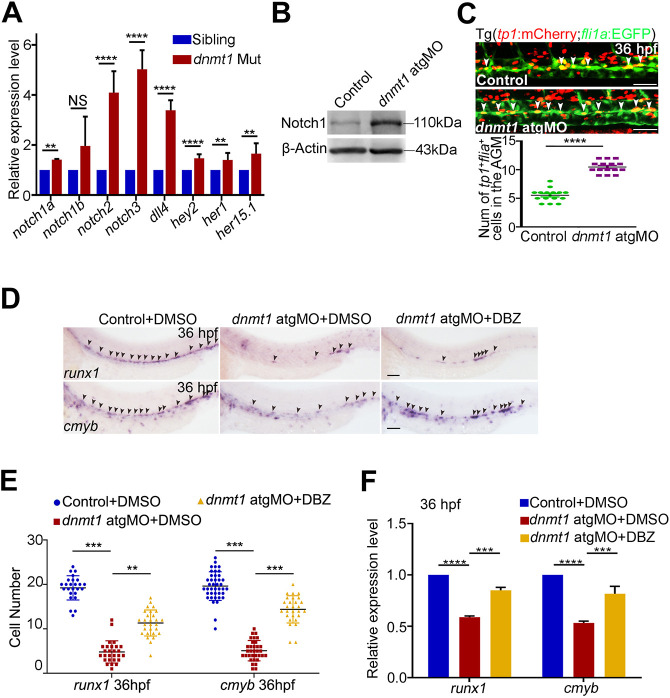
**Dnmt1 represses Notch signaling to regulate HSPC generation.** (A) qPCR analysis showing expression of Notch-related genes *notch1a*, *notch1b*, *notch2*, *notch3*, *dll4*, *hey2*, *her1* and *her15.1* in *kdrl*^+^/*runx1*^+^ HECs. *n*=3 replicates. (B) Protein level of Notch1 in control and *dnmt1*-deficient embryos at 36 hpf. (C) Confocal imaging showing the number of *tp1^+^/fli1a^+^* cells in the AGM in control and *dnmt1* morphants (white arrowheads, upper panels) with quantification (bottom panel). *n*≥3 replicates. (D) WISH analysis showing the expression of *runx1* and *cmyb* (arrowheads) at 36 hpf in control and *dnmt1* morphants treated with DMSO or DBZ. *n*≥3 replicates. (E) Statistical analysis of the WISH data in D. (F) qPCR analysis of *runx1* and *cmyb* expression in control and *dnmt1* morphants treated with DMSO or DBZ at 36 hpf. *n*=3 replicates. Data are mean±s.d. **P<0.01, ***P<0.001, ****P<0.0001 (unpaired two-tailed Student's *t*-test). NS, no significance. Scale bars: 50 µm (C); 100 µm (D).

Given that regulators of arterial fate, including Notch signaling, are implicated in HSPC generation from HEC in zebrafish (Zhang et al., 2017; Zhang et al., 2015), together with the finding that specifically methylated regions in HECs are enriched in Notch signaling (Fig. 3B), we further explored the link between Notch signaling and DNA methylation during HSPC generation. First, we examined the expression of a panel of Notch-related genes by qPCR in the HECs of sibling and *dnmt1* mutant embryos at 36 hpf. A significant induction in the expression of several Notch-related genes was observed in the *dnmt1* mutant (Fig. 5A). Western blotting showed that the protein levels of Notch1 were higher in *dnmt1*-deficient embryos compared with that in siblings (Fig. 5B). Next, we detected Notch activity *in vivo* using Tg(*tp1*:mCherry/*fli1a*:EGFP) embryos (tp1:mCherry expresses mCherry protein driven by the promoter of terminal protein 1 gene containing the Notch-responsive element), which could specifically indicate Notch activity in ECs, and observed an increased number of *tp1*^+^ ECs upon *dnmt1* knockdown compared with control embryos (Fig. 5C), suggesting that Notch activity was increased in ECs. We then investigated whether inhibition of Notch signaling could rescue HSPC defects in *dnmt1*-deficient embryos. We performed DBZ (a Notch inhibitor) treatment from 26 hpf to specifically inhibit Notch signaling during HSPC generation (Zhang et al., 2015) and found that the diminished expression of *cmyb* and *runx1* in *dnmt1*-deficient embryos could be efficiently restored (Fig. 5D,E), which was also confirmed by qPCR (Fig. 5F). The increased Notch1 protein level and impaired HSPC generation were also restored by DBZ treatment in *dnmt1* sMO-injected embryos (Fig. S6A-C). Collectively, these results demonstrate that, in the absence of Dnmt1, upregulation of Notch signaling is responsible for the observed HSPC defects.

**Fig. 6.**
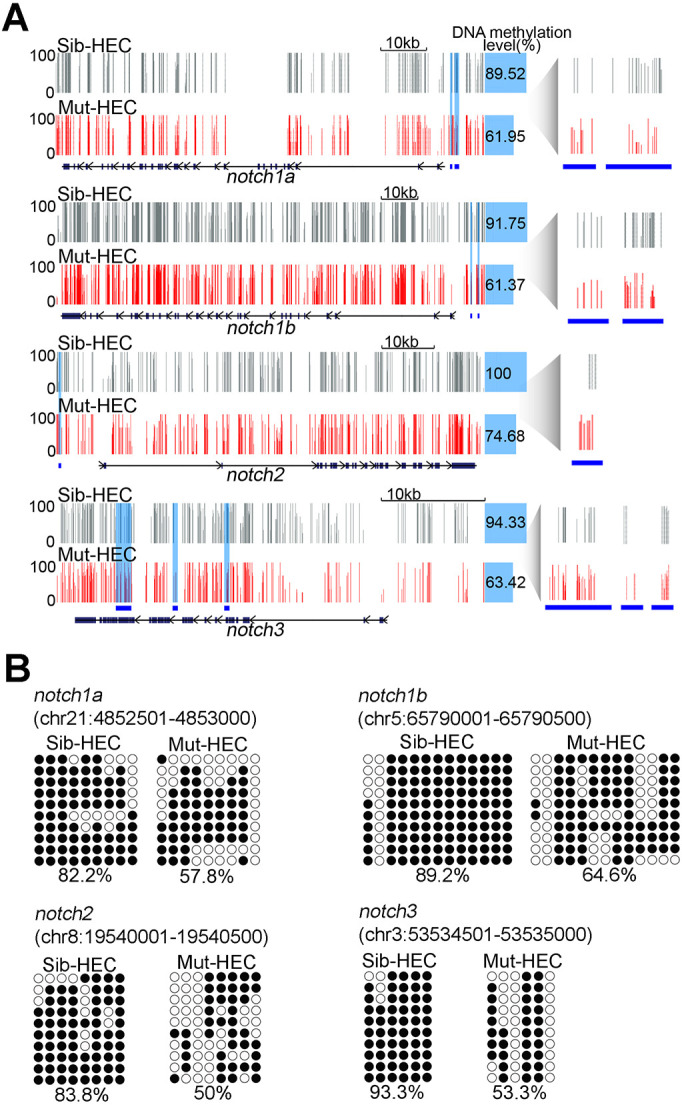
**Loss of Dnmt1 reduces methylation levels of Notch genes.** (A) Representative display of DNA methylation at Notch signaling-related genes in HECs in sibling and *dnmt1* mutants. Blue shading and blue lines show DMRs. Each vertical line on tacks represents a 5mC site. (B) Bisulfite sequencing analysis of DNA methylation at DMRs in *notch1a*, *notch1b*, *notch2* and *notch3* in HECs in sibling and *dnmt1* mutants. Filled circle, methylated CpG; unfilled circle, unmethylated CpG. The number underneath each sequencing diagram indicates the percentage of all methylated CpG sites over the total number of CpG sites of the sequenced colonies for each sample.

Finally, we investigated the relationship between methylation level and repression of a large panel of Notch signaling genes. We explored the WGBS data of HECs in sibling and *dnmt1* mutants, and found that most of Notch genes, including *notch1a*, *notch1b*, *notch2* and *notch3*, displayed hypo-methylation at proximal elements in the *dnmt1* mutant compared with controls (Fig. 6A). Direct sequencing of bisulfite-PCR products confirmed the reduced methylation levels in these regulatory regions of Notch genes in HECs upon *dnmt1* knockout (Fig. 6B). Taken together, these results support the role of Dnmt1-mediated methylation in regulation of HSPC generation through repression of Notch genes (Fig. S6D).

## DISCUSSION

The process of HSPC generation from arterial ECs is accompanied by alterations in the global transcriptome, epigenome and 3D genome topology (Wu and Hirschi, 2021). Using multi-omics approaches and bioinformatic analysis, the landscape of HSPC development has been systemically established at transcriptomic and epigenomic levels, including the DNA methylome (Baron et al., 2018; Hou et al., 2020; Li et al., 2021; Vink et al., 2020; Zeng et al., 2019; Zhu et al., 2020). However, the dynamic epigenetic regulatory mechanisms underlying HSPC development remain largely unclear in any given vertebrate model. To this end, in the present study we determined the functional role of DNA methylation during HSPC generation by depleting endogenous *dnmt1* in zebrafish. We revealed a dynamic DNA methylome in HSPC generation in normal development, and also demonstrated the functional role of DNA methylation during EHT, in which the repression of arterial genes and Notch signaling is a key step. Notably, a recent study described a DNA methylation landscape during mouse HSC development, including the endothelial-to-pre-HSC transition, and reported that the endothelial-featured genes undergo gain-of-methylation in T1 pre-HSCs (Li et al., 2021), which is consistent with our findings in zebrafish. Our functional results in zebrafish, together with the dynamic methylome in zebrafish and mice, demonstrate the evolutionary conservation of DNA methylation as a repressive regulatory mechanism for EHT in vertebrates.

Our finding also showed that DMRs displaying demethylation during ECs to HSPCs transition were mainly enriched in hematopoiesis-related terms as shown in Cluster 6 in Fig. 1C. Consistently, a very recent study reported that Tet-mediated DNA demethylation in mice could activate hematopoietic programs in ECs during HSPC specification through demethylation of NFκB1 and master hematopoietic transcription factors (such as Gata1/2, Runx1 and Gli1b). Global or endothelial-specific loss of Tet enzymes disrupted HSPC development (Ma et al., 2022). These findings demonstrated the regulatory role of DNA methylation and demethylation during HSPC generation. Combined with our findings here, we propose that Dnmt1-mediated methylation of endothelial genes and Tet-mediated demethylation of hematopoietic genes coordinately protect HSPC generation during embryonic hematopoiesis.

The Notch signaling pathway is highly conserved, and plays essential roles during embryogenesis, including artery-vein specification and definitive hematopoiesis (Fang and Hirschi, 2019; Krebs et al., 2000; Kumano et al., 2003; Lawson et al., 2001; Lin et al., 2007; Robert-Moreno et al., 2005; Swift and Weinstein, 2009). Activation of the Notch pathway in endothelial cells could lead to the expression of downstream arterial markers, which is sufficient and essential for arteriovenous specification (Fang and Hirschi, 2019; Lin et al., 2007; Swift and Weinstein, 2009). Subsequently, Notch signaling should be tuned down in HECs to facilitate HSPC emergence (Gama-Norton et al., 2015; Lizama et al., 2015; Zhang et al., 2015). The precise control of Notch activity is largely unknown, but extremely important during definitive hematopoiesis. Previous studies have shown that receptor-ligand interactions determine the strength of Notch signaling, demonstrating that a high-strength Dll4 signal promotes the arterial fate, whereas a low-strength Jag1 signal leads to the specification of HSCs (Gama-Norton et al., 2015). Besides, downregulation mechanisms of Notch signaling were found to depend on the degradation of Notch receptors, such as the Gpr183-induced proteasome pathway and Blos2-mediated endolysosomal degradation of Notch1a (Zhang et al., 2015; Zhou et al., 2016). Another different layer of downregulation of Notch signaling involves m^6^A-induced mRNA decay (Zhang et al., 2017). In the present study, we showed that DNA methylation modulates HSPC generation through repression of Notch signaling transcriptional activity. We found that higher expression of Notch signaling genes with hypo-methylated regions led to the increased Notch signaling in HECs, thereby inhibiting EHT. Interestingly, a recent study demonstrated that Dnmt1-mediated global methylation can reset an epigenetic gate to safeguard embryonic development against premature activation of adult programs (Wu et al., 2021 preprint). In the present work, the DNA methylome might play a similar role as a rheostat to effectively repress the arterial program and promote HSPC generation.

Based on the present and previous findings, we propose that Notch signaling is modulated at different levels by integrating DNA methylation, transcriptional control (Corada et al., 2013; Lizama et al., 2015), RNA modification (Zhang et al., 2017) and protein stability (Zhang et al., 2015; Zhou et al., 2016) during EHT, suggesting the existence of complex Notch regulatory mechanisms underlying HSPC generation. The multi-dimensional regulatory mode of gene expression may help to efficiently and accurately regulate the activity of key factors in the instantaneous process, as a cell fate switch. And such complex regulatory machinery at different levels during biological processes is not rare. For example, the maternal-to-zygotic transition (MZT), which involves maternal RNA and protein decay and zygotic genome activation, is an important event during early embryogenesis across vertebrates (Yartseva and Giraldez, 2015). RNA modification (including uridylation and m^6^A), miR-430 and codon usage have been reported to all facilitate maternal mRNA decay (Bazzini et al., 2012; Chang et al., 2018; Giraldez et al., 2006; Laue et al., 2019; Mishima et al., 2012; Zhao et al., 2017). The core regulatory machinery of epithelial-to-mesenchymal transition (EMT) is also governed by multiple genetic and epigenetic regulators, such as transcription factors TWIST1 and SNAIL1, multiple miRNAs (miR-200) and post-translational modifications, including ubiquitylation and phosphorylation (Nieto et al., 2016).

In summary, we provide strong evidence that DNA methylation-mediated epigenetic modification facilitates HSPC generation through the repression of Notch signaling. This finding deepens our understanding of HSPC specification, which may help in designing new strategies for HSPC generation *in vitro*.

## MATERIALS AND METHODS

### Zebrafish strains

Zebrafish strains including Tübingen, Tg(*kdrl*:mCherry) (Bertrand et al., 2010), Tg(*cmyb*:EGFP) (North et al., 2007), Tg(*runx1*:en-GFP) (Zhang et al., 2015), Tg(*tp1*:mCherry) (Parsons et al., 2009), Tg(*fli1a*:EGFP) (Lawson and Weinstein, 2002) and *dnmt1* mutant (Anderson et al., 2009) were raised at 28.5°C in system water (conductivity at 500∼550 μs/cm and pH 7.0∼7.5). Zebrafish embryos were obtained via the natural spawning of adult zebrafish. This study was approved by the Ethical Review Committee of the Institute of Hematology, Chinese Academy of Medical Sciences, China.

### Morpholinos, mRNA synthesis and plasmid construction

The antisense MOs including *dnmt1* atgMO and *dnmt1* splice MO were purchased from Gene Tools. The MO sequences were: *dnmt1* atgMO, 5′-ACAATGAGGTCTTGGTAGGCATTTC-3′ (Rai et al., 2006); *dnmt1* splice MO (sMO), 5′-AGGTCTTGGTAGGCATTTCAAGTTC-3′ (Wang et al., 2017). The full-length mismatched *dnmt1* mRNA and truncated-mismatched *dnmt1* mRNA without methyltransferase domain were generated using SP6 mMessage Machine kit (AM1340, Ambio). The full length coding DNA sequence of mismatched *dnmt1* was cloned into pDestTol2pA2 using the *hsp70* or *fli1a* promoter and an EGFP reporter using HiFi DNA Assembly Master Mix (E2621S, NEBuilder). MOs, mRNA and constructs with *tol2* mRNA were injected into zebrafish embryos at one-cell stage.

### WISH and FISH

WISH was performed as previously described with probes, including *cmyb*, *runx1*, *gata1*, *pu.1*, *ae1-globin*, *l-plastin*, *rag1*, *dll4*, *ephrin-B2a*, *dltC*, *hey2* and *dnmt1* (Wang et al., 2013; Zhang et al., 2017). FISH was performed as previously described (Heng et al., 2020) with *dnmt1*, *cmyb/runx1* and *egfp* probes.

### Western blotting

Protein was extracted from the dissected trunk regions of 36 hpf zebrafish embryos. Western blotting was performed as previously reported (Zhang et al., 2014) using the following antibodies: anti-β-Actin antibody (4967, Cell Signaling Technology, 1:1000), anti-Notch1 antibody (ab65297, Abcam, 1:100), anti-Dnmt1 antibody (24206, Proteintech, 1:300), anti-Runx1 antibody (AS-55593, Ana Spec, 1:200).

### Quantitative real-time PCR

Total RNA was extracted from the dissected trunk regions of 36 hpf zebrafish embryos using TRIzol reagent (15596018, Life Technologies) or from sorted cells using RNeasy Micro Kit (74004, Qiagen). The mRNA was reverse transcribed using M-MLV Reverse Transcriptase (M1701, Promega). qPCR was performed (Zhang et al., 2017) using premixture (FP205-03, Tiangen) on a CFX96 Real Time PCR system (Bio-Rad). Primers are listed in Table S1.

### Chemical treatment

Zebrafish embryos were treated with 4 μM Notch inhibitor DBZ (SML0649, Sigma-Aldrich) from 26 hpf to 36 hpf. Embryos were treated with 10 μM 5-Aza (A2385, Sigma-Aldrich) from 24 hpf to 36 hpf.

### Confocal imaging

The transgenic zebrafish embryos were manually dechorionated and placed in 1% low-melting-point agarose. Microscopy was performed using an Andor Dragonfly 505 confocal microscope (Oxford Instruments). The images were edited using ImageJ (National Institutes of Health) and Photoshop CS6 (Adobe).

### Bisulfite conversion and sequencing

Hemogenic endothelial cells (*kdrl*:mCherry^+^/*runx1*:en-GFP^+^) from trunk region in siblings and *dnmt1* mutants were sorted using flow cytometry (MoFlo XDP, Beckman Coulter) for direct digestion using EZ DNA Methylation-Direct™ Kit (D5020, Zymo). Digested material was bisulfite-converted following manual instructions. Bisulfite-converted DNA was amplified using primers designed by online tool Bisulfite Primer Seeker (https://www.zymoresearch.com/). ZymoTaq PreMix (E2003, Zymo) was used for amplifying bisulfite-converted DNA. Then PCR products were gel-purified, cloned into pGEM-T vector (A362A, Promega) and sequenced. The sequencing results were analyzed using online tool QUMA (http://quma.cdb.riken.jp/) (Kumaki et al., 2008). Primers for bisulfite sequencing are listed in Table S2.

### Cell transplantation

Donor embryos were first co-injected with Rhodamine-dextran (D1841, Molecular Probes) together with *dnmt1* atgMO. Then, at shield stage, ventral mesodermal cells (30-50 cells) from donor embryos were transplanted into the corresponding regions of host embryos. Host embryos were raised until 36 hpf, then microscopic observation and photography were carried out using an Andor Dragonfly 505 confocal microscope (Oxford Instruments).

### Whole genome bisulfite sequencing

Zebrafish ECs, HECs and HSPCs from the trunk region of 36 hpf Tg(*kdrl*:mCherry/*runx1*:en-GFP) embryos were sorted as previously reported (Zhang et al., 2015). For WGBS, cells from siblings and *dnmt1* mutants were resuspended in PBE buffer with DAPI (1 μg/ml, Sigma-Aldrich). Library conduction was performed according to the previous protocol (Smallwood et al., 2014). We sorted 500 cells into 10 μl M-digestion buffer (D5044, Zymo). Sample digestion, bisulfite conversion and DNA purification were performed using EZ-96 DNA Methylation-Direct MagPrep kit (D5044, Zymo) with the following modifications: samples were incubated at 50°C for 3 h, and proteinase K was inactivated by incubation at 75°C for 30 min, then bisulfite conversion was followed by incubation at 98°C for 8 min, 64°C for 3.5 h and 4°C for 20 h. DNA was eluted in 42.5 μl M-Elution Buffer for the purification procedure, combined with 1× Blue Buffer (P7010-HC-L, Enzymatics), 0.2 mM dNTPs (R0193, Fermentas), 1 μM oligo1 (CTACACGACGCTCTTCCGATCTNNNNNN) (a total of 49 μl) before being incubated at 65°C for 3 min and transferred to ice. Then, 1 μl of 50 U/μl Klenow fragment (3′-5′ exo-) (P7010-HC-L, Enzymatics) was added for the first-strand DNA synthesis followed by incubation at 4°C for 5 min, raised to 37°C at a rate of 1°C/15 s, kept at 37°C for 90 min and an additional 10 min at 70°C to inactivate the enzyme activity. Samples were then incubated with 2 μl of 20 U/μl exonuclease I (M0293S, New England Biolabs) and 48 μl nuclease-free water (4387936, Ambion) at 37°C for 60 min before DNA was purified using 0.8× AMPure XP beads (A63882, Beckman Coulter) according to the manufacturer's instructions. DNA was eluted in 42.5 μl nuclease-free water, then 1× Blue Buffer, 0.2 mM dNTPs and 1 μM oligo2 (AGACGTGTGCTCTTCCGATCTNNNNNN) were added (a total of 49 μl). Following incubation at 98°C for 2 min and immediate transferral to ice, the second-strand DNA was synthesized by adding 1 μl Klenow fragment to the solution and incubation at 4°C for 5 min, raising to 37°C at a rate of 1°C/15 s, keeping at 37°C for 90 min. The synthesized DNA was then purified using 0.8× AMPure XP beads and eluted in 22 μl nuclease-free water, and combined with 25 μl of 2× KAPA HiFi HotStart ReadyMix (KK2602, KAPA), 1.5 μl universal PCR primer and 1.5 μl index primer (KK8504, KAPA) for library amplification with the following PCR steps: 95°C 3 min, 12 cycles of (98°C 20 s, 65°C 30 s, 72°C 1 min), 72°C 1 min, 4°C hold. Amplified libraries were purified twice using 0.8× AMPure XP beads. Finally, the library was sequenced on an Illumina NovaSeq 6000 system (Novogene).

### Analysis of whole genome bisulfite sequencing data

Raw sequencing reads were first trimmed to remove adapters and law quality bases by using Trim Galore (v0.6.6). Next retained reads were aligned to zebrafish reference genome (GRCz11) using BS-Seeker2 (v2.1.8) (Guo et al., 2013), and SAMtools (v1.11) was used to remove PCR duplicates (Li et al., 2009). To estimate the DNA methylation levels, we first divided the genome into consecutive 500 bp tiles and the methylation values were calculated by the number of methylated cytosines divided by the total number of methylated and unmethylated cytosines reported by sequencing data. The mean methylation level of every 500 bp tiles was measured on behalf of the genome-wide DNA methylation level. Tiles were defined as DMR if they showed a difference of methylation levels larger than 0.2 and adjusted *P*-value less than 0.05 between two groups (unpaired two-tailed Student's *t*-test). *P*-values were adjusted using the Benjamini–Hochberg correction. Only CpG sites recovered by both groups were considered to perform DMR analysis. To evaluate the enrichment of DMRs on different genomic regions, we defined promoter regions as upstream 1 kb to downstream 1 kb of transcription start sites (TSS), gene bodies as regions from TSS to transcription end sites and downloaded genomic annotations of exons, LTRs, LINEs, SINEs, Introns and CpG island regions from the University of California Santa Cruz (UCSC) genome browser (GRCz11). DMR enrichment analysis was performed using LOLA (v1.20.0) (Sheffield and Bock, 2016). GREAT was used to annotate DMRs. The sequencing metrics for WGBS data, statistical summary of mCG sites and statistical summary of CH sites are listed in Tables S3, S4 and S5, respectively.

### RNA-seq and data sequencing

Equal amounts (500 cells) of ECs, HECs and HSPCs from the trunk region of 36 hpf Tg(*kdrl*:mCherry/*runx1*:en-GFP) siblings and *dnmt1* mutants were sorted and collected. RNA-seq library construction for low input cells was carried out as previously described (Picelli et al., 2014; Zhu et al., 2021). The library was sequenced on an Illumina NovaSeq 6000 system (Novogene). After removing low quality reads and adapters using cutadapt (v1.18) (Martin, 2011), the clean reads were mapped to a *Danio rerio* reference genome (GRCz11) using hisat2 (v2.2.1) (Kim et al., 2019). The gene expression level was quantified using HTSeq (v0.12.4) (Anders et al., 2015) and differentially expressed genes (DEGs) were determined by DESeq2 (v1.30.1) (Love et al., 2014) requiring adjusted *P*-value<0.5 and |log2FC|≥1. GO analysis was performed using R package clusterProfiler (v3.18.1) (Yu et al., 2012) and GSEA was performed as previously described (Subramanian et al., 2005). The sequencing metrics for RNA-seq data are listed in Table S6.

### Integrated analysis of DNA methylation and RNA transcription data

To resolve the potential regulation of gene expression dynamics by DNA methylation, we performed correlation analysis between gene expression values and promoter methylation levels of corresponding genes in sibling samples and mutant samples. In detail, we first defined DEGs. Then we calculated Pearson correlations between gene expression and promoter methylation of each DEG. Genes with strong negative correlations (correlation<−0.3) in sibling samples rather than mutant samples were assumed to be under the regulation of DNA methylation. Genes as samples with decreased expression and promoters with increased methylation levels from EC to HEC and from HEC to HSPC are listed in Table S7.

### Statistical analysis

The quantification of WISH results was processed by ImageJ referring to previous reported protocol (Dobrzycki et al., 2020). All data were presented as mean±s.d. and analyzed with GraphPad PRISM 8 software. All statistical evaluation was performed for at least three independent biological repeats. The unpaired two-tailed Student's *t*-test was performed for single statistical investigations between two groups. A two-way analysis of variance (ANOVA) was carried out using the Sidak correction for multiple comparisons. *P*-values were used for significance evaluation, and significance levels are shown as *P<0.05, **P<0.01, ***P<0.001, ****P<0.0001. N.S. indicates non significance.

## Supplementary Material

Supplementary information

Reviewer comments
