# Supplementary material for: DNA methylation safeguards the generation of hematopoietic stem and progenitor cells by repression of Notch signaling
Source: Development. 2022 May 25;149(10):dev200390. doi: 10.1242/dev.200390 (PMC9188753; doi:10.1242/dev.200390)
Supplement: Supplementary information [file develop-149-200390-s1.pdf]

sFig1.

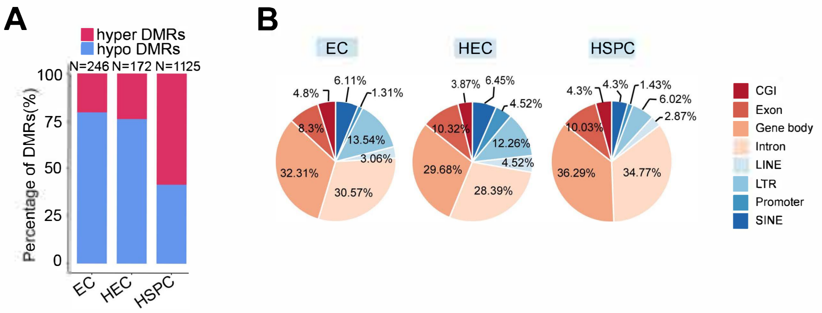

Fig. S1. Dynamic DNA methylome during EHT.

The amount of hypermethylated DMRs (hyper DMRs) and hypomethylated DMRs (hypo DMRs) identified in EC, HEC and HSC, respectively.

The genome distribution of DMRs that are specific in EC, HEC and HSPC, respectively.

Development • Supplementary information

sFig2.

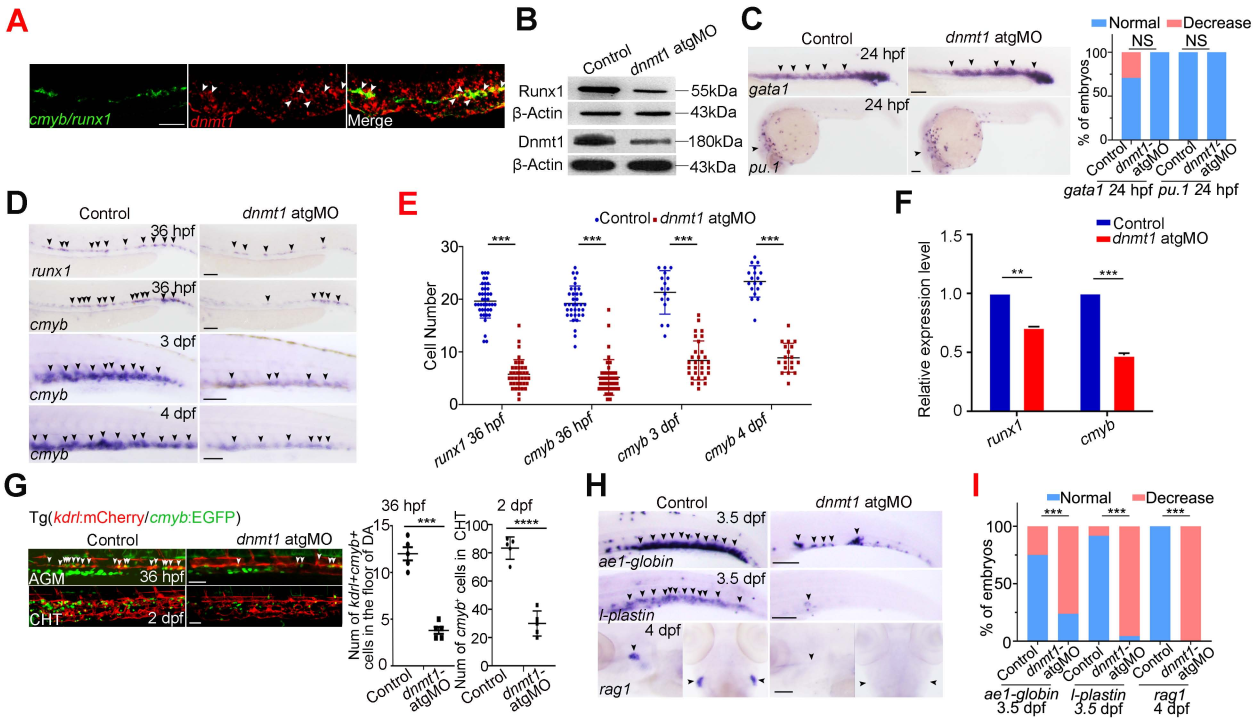
A
cmyb/runx1
dnmt1
Merge
B
Control
dnmt1 atgMO
Runx1
β-Actin
Dnmt1
β-Actin
55kDa
43kDa
180kDa
43kDa
C
Control
dnmt1 atgMO
24 hpf
gata1
pu.1
Normal
Decrease
NS
% of embryos
gata1 24 hpf
pu.1 24 hpf
D
Control
dnmt1 atgMO
36 hpf
runx1
cmyb
3 dpf
4 dpf
E
Control
dnmt1 atgMO
Cell Number
runx1 36 hpf
cmyb 36 hpf
cmyb 3 dpf
cmyb 4 dpf
F
Control
dnmt1 atgMO
Relative expression level
runx1
cmyb
G
Tg(kdrl:mCherry/cmyb:EGFP)
Control
dnmt1 atgMO
AGM
36 hpf
CHT
2 dpf
Num of kdrl+cmyb+ cells in the floor of DA
Num of cmyb+ cells in CHT
H
Control
dnmt1 atgMO
3.5 dpf
ae1-globin
l-plastin
4 dpf
rag1
I
Normal
Decrease
% of embryos
ae1-globin 3.5 dpf
l-plastin 3.5 dpf
rag1 4 dpf

Fig. S2. Defects in HSPC development in *dnmt1* morphants.

(A) Double fluorescence *in situ* hybridization (FISH) analysis showing co-expression of *dnmt1* with *cmyb*/*runx1* (white arrowheads). Scale bars, 30 µm.

(B) Western blotting analysis showing protein levels of Runx1 and Dnmt1 in control and *dnmt1* morphants at 36 hpf.

(C) Expression of the erythroid marker *gata1* and the myeloid marker *pu.1* at 24 hpf in control and *dnmt1*-deficient embryos (left panel) and statistical analysis (right panel). Scale bar, 100 µm. The arrowheads indicate the expression of *gata1* and *pu.1*. n⩾3 replicates. student's *t* test, NS, no significance.

(D) WISH analysis showing the expression of *runx1* and *cmyb* in the AGM at 36 hpf, and *cmyb* expression in CHT region at 3 dpf and 4 dpf, in control and *dnmt1*-deficient embryos, respectively. Scale bar, 100 µm. The arrowheads indicate the expression of *runx1* and *cmyb*. n⩾3 replicates.

(E) Statistical analysis of the WISH data in (D). Error bars, mean ± SD, ***P < 0.001, student's *t* test.

(F) qPCR analysis of *runx1* and *cmyb* expression in control and *dnmt1* morphants at 36 hpf. Error bars, means ± SD, n=3 replicates, **P < 0.01, ***P < 0.001, student's *t* test.

(G) Confocal imaging showing the number of *kdrl*$^{+}$/*cmyb*$^{+}$ HECs in AGM region at 36 hpf (white arrowheads) and *cmyb*$^{+}$ HSPCs in CHT region at 2 dpf, in control and *dnmt1* morphants with quantification. Error bars, means ± SD, ***P < 0.001, ****P < 0.0001, student's *t* test. Scale bars, 50 µm. n⩾3 replicates.

(H) WISH analysis showing the expression of *ae1-globin* and *l-plastin* in the CHT region at 3.5 dpf and *rag1* in the thymus at 4 dpf, in control and *dnmt1* morphants. Scale bar, 100 µm. The arrowheads indicate the expression of corresponding hematopoietic genes. n⩾3 replicates.

(I) Statistical analysis of the WISH data in (H). Error bars, mean ± SD, ***P < 0.001, student's *t* test.

Development • Supplementary information

sFig3.

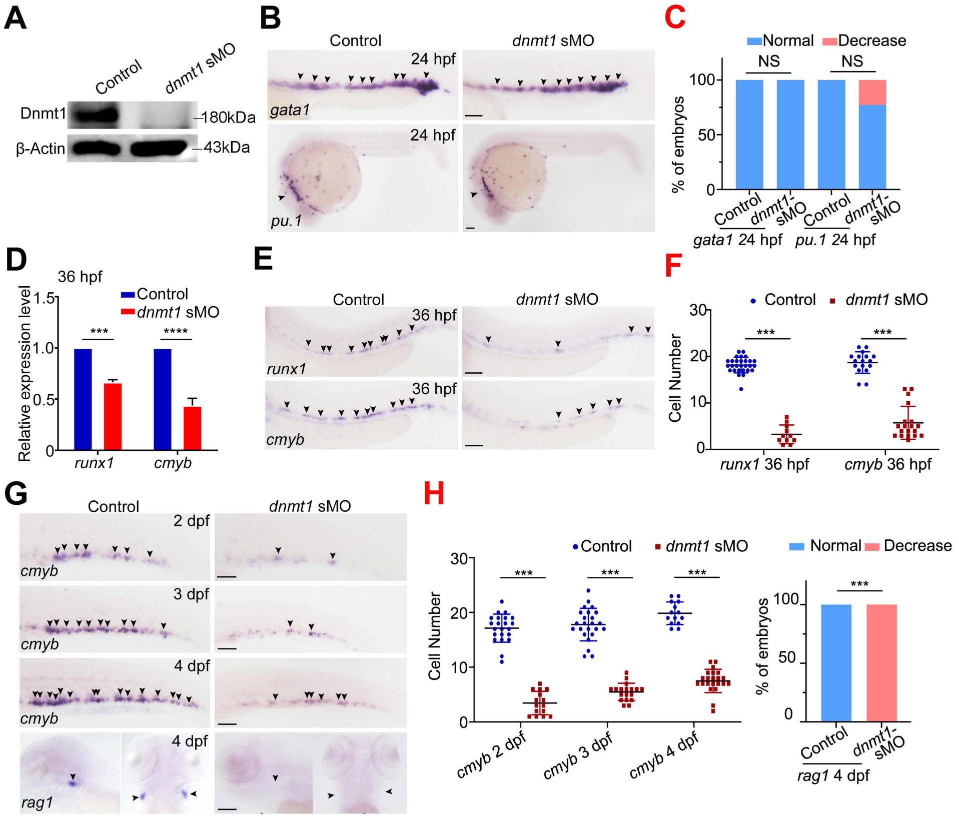

Development • Supplementary information

Fig. S3. HSPC development is impaired in *dnmt1* sMO-injected embryos.

(A) Protein level of Dnmt1 in control and *dnmt1* sMO-injected embryos at 36 hpf.

(B) Expression of the erythroid marker *gata1* and the myeloid marker *pu.1* in control and *dnmt1* sMO-injected embryos at 24 hpf. Scale bar, 100 µm. The arrowheads indicate the expression of *gata1* and *pu.1*. n⩾3 replicates.

(C) Statistical analysis of the WISH data in (B). Error bars, mean ± SD, NS, no significance, student's *t* test.

(D) qPCR analysis of *runx1* and *cmyb* expression in control and *dnmt1* sMO-injected embryos at 36 hpf. Error bars, means ± SD, ***P < 0.001, ****P < 0.0001, student's *t* test.

(E) Expression of *runx1* and *cmyb* in the AGM region at 36 hpf in control and *dnmt1* sMO-injected embryos. Scale bar, 100 µm. The arrowheads indicate the expression of *runx1* and *cmyb*. n⩾3 replicates.

(F) Statistical analysis of the WISH data in (B). Error bars, mean ± SD, ***P < 0.001, student's *t* test.

(G) WISH analysis showing expression of *cmyb* in the CHT region at 2 dpf, 3 dpf and 4 dpf, and *rag1* in the thymus at 4 dpf, in control and *dnmt1* sMO-injected embryos, respectively. Scale bar, 100 µm. The arrowheads indicate the expression of *cmyb* and *rag1*. n⩾3 replicates.

(H) Statistical analysis of the WISH data in (G). Error bars, mean ± SD, ***P < 0.001, student's *t* test.

Development • Supplementary information

sFig 4.

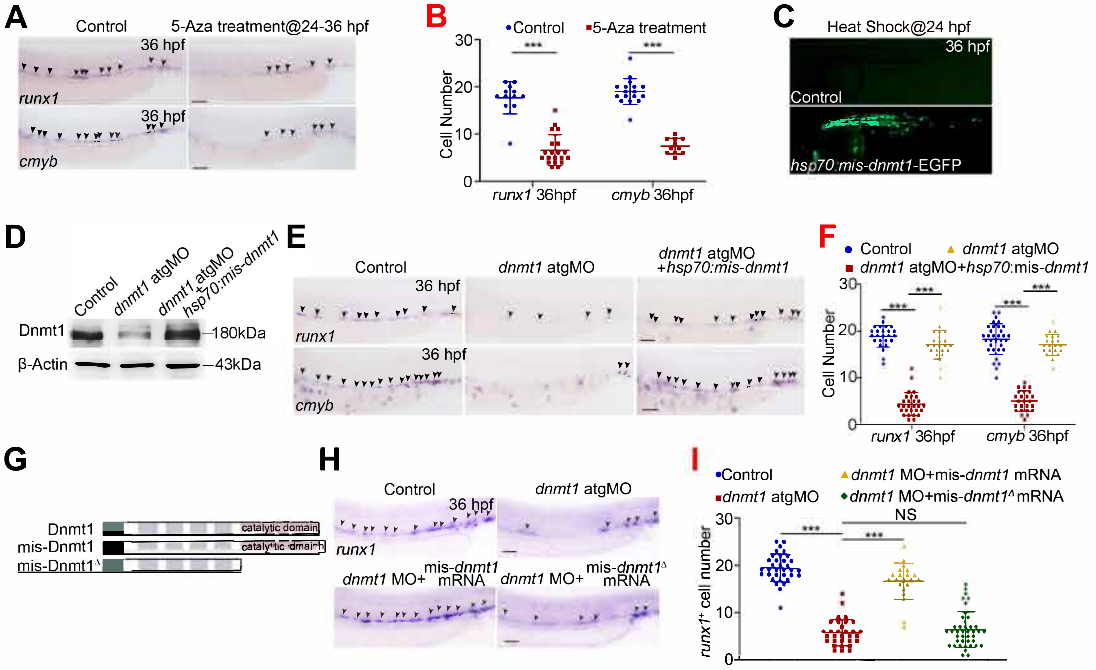

Development • Supplementary information

Fig. S4. DNA methylation is required during EHT.

(A) Expression of *runx1* and *cmyb* in the AGM region at 36 hpf in control embryos and 5-Aza-treated embryos. Treatment was performed from 24 to 36 hpf. Scale bar, 100 µm. The arrowheads indicate the expression of *runx1* and *cmyb*. n⩾3 replicates.

(B) Statistical analysis of the WISH data in (A). Error bars, mean ± SD, ***P < 0.001, student's *t* test.

(C) The EGFP expression in embryos-injected with *hsp70*:mis-*dnmt1*-EGFP construct at 36 hpf. Heat shock was performed at 24 hpf.

(D) Protein level of Dnmt1 in control, *dnmt1* morphants, and embryos-coinjected with *dnmt1* atgMO and *hsp70*:mismatch-*dnmt1*-EGFP constructs at 36 hpf.

(E) Expression of *runx1* and *cmyb* in control, *dnmt1* morphants, and embryos-coinjected with *dnmt1* atgMO and *hsp70*:mismatch-*dnmt1*-EGFP constructs. Scale bar, 100 µm. The arrowheads indicate the expression of *runx1* and *cmyb*. n⩾3 replicates.

(F) Statistical analysis of the WISH data in (E). Error bars, mean ± SD, ***P < 0.001, student's *t* test.

(G) The schematic structures of *dnmt1* mRNA, *dnmt1* mis-mRNA and truncated *dnmt1* mis-mRNA.

(H) WISH analysis of *runx1* expression in control, *dnmt1* morphants, *dnmt1* morphants with overexpression of *dnmt1* mis-mRNA or truncated *dnmt1* mis-mRNA (mis-*dnmt1*$^{\triangle}$ mRNA), respectively. Scale bars, 100 µm. The arrowheads indicate the expression of *runx1*. n⩾3 replicates.

(I) Statistical analysis of the WISH data in (H). Error bars, mean ± SD, ***P < 0.001, NS, no significance, student's *t* test.

Development • Supplementary information

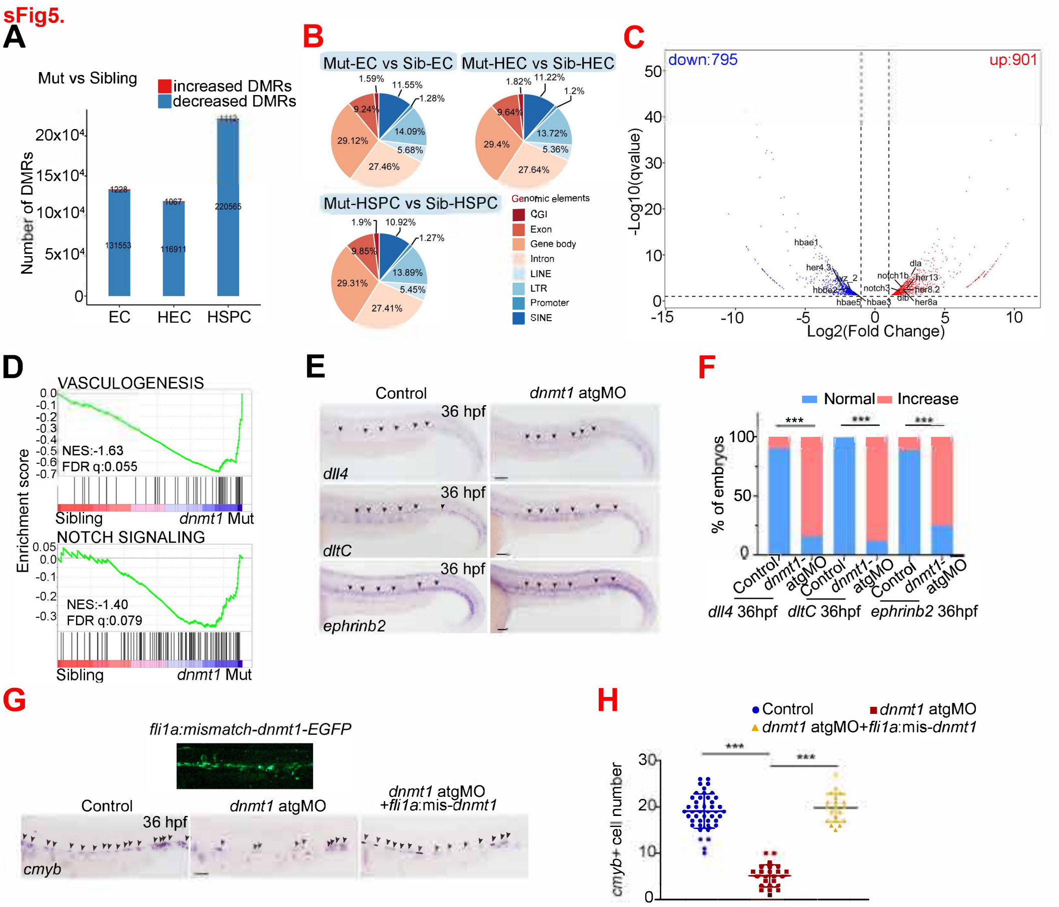

Fig. S5. The expression of arterial genes is increased in *dnmt1* morphants.

(A) The number of DMRs comparing sibling and *dnmt1* mutant samples in EC, HEC and HSPC.

(B) Genome distribution of DMRs identified in (A).

(C) Volcano plots for differential expressed genes identified by RNA-seq in *dnmt1* mutant compared with siblings in HSPC.

Development • Supplementary information

(D) Gene set enrichment analysis (GSEA) of genes associated with vasculogenesis and Notch signaling in HEC comparing sibling and *dnmt1* mutant samples.

(E) WISH analysis showing the expression of arterial genes *dll4*, *dltC* and *hey2* in control and *dnmt1* morphants at 36 hpf (arrowheads). Scale bar, 100 µm. n⩾3 replicates.

(F) Statistical analysis of the WISH data in (E). Error bars, mean ± SD, ***P < 0.001, student's *t* test.

(G) Expression of *cmyb* expression (arrowheads) in control, *dnmt1* morphants, and embryos-coinjected with *dnmt1* atgMO and *fli1a*:mismatch-*dnmt1*-EGFP constructs (Bottom panel). Upper panel, the embryos-injected with *fli1a*:mis-*dnmt1*-EGFP construct showing vessel-specific EGFP expression at AGM region. Scale bar, 100 µm. n⩾3 replicates.

(H) Statistical analysis of the WISH data in (G). Error bars, mean ± SD, ***P < 0.001, student's *t* test.

Development • Supplementary information

sFig6.

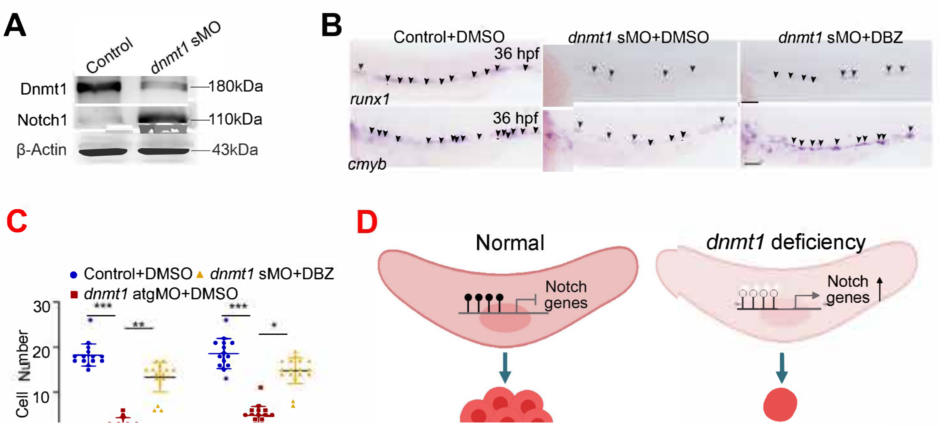

Fig. S6. Notch activity is elevated in dnmt1 sMO-injected embryos.

(A) Protein levels of Notch1 in control and *dnmt1* sMO-injected embryos at 36 hpf.

(B) Expression of *runx1* and *cmyb* in control and *dnmt1* sMO-injected embryos treated with DMSO or DBZ, respectively. Scale bar, 100 μm. The arrowheads indicate the expression of *runx1* and *cmyb*. n⩾3 replicates.

(C) Statistical analysis of the WISH data in (B). Error bars, mean ± SD, *P < 0.05, **P < 0.01, ***P < 0.001, student's *t* test.

(D) Model depicting the role of Dnmt1-mediated methylation in regulation of HSPC generation through repression of Notch genes. During normal development (left panel), Notch signaling is tightly controlled by DNA methylation to ensure HSPC production. In the absence of *dnmt1* (right panel), Notch signaling is abnormally activated due to the hypomethylation, thereby causing impaired HSPC generation.

Development • Supplementary information

Table S1. The primers for QPCR.

Click here to download Table S1

Table S2. The primers for bisulfite sequencing.

Click here to download Table S2

Table S3. The sequencing metrics for WGBS data.

Click here to download Table S3

Table S4. The statistical summary of mCG sites.

Click here to download Table S4

Table S5. The statistical summary of CH sites.

Click here to download Table S5

Table S6. The sequencing metrics for RNA-seq data.

Click here to download Table S6

Table S7. Genes with decreased expression and promoter with increased methylation levels from EC to HEC and from HEC to HSPC.

Click here to download Table S7

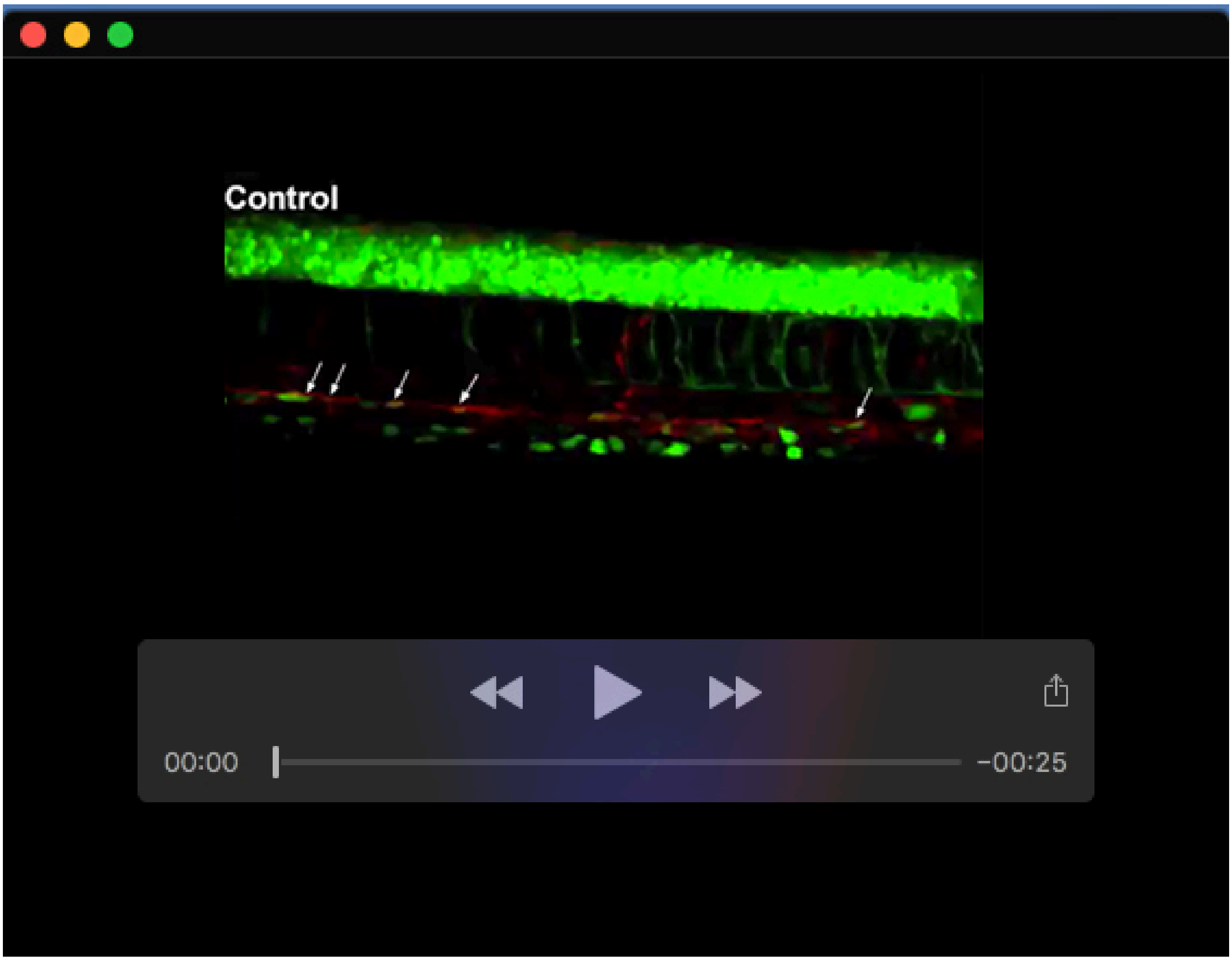

Movie 1. Time-lapse lineage tracing of EHT in control embryos.

The fate transition via EHT was observed in control Tg(*kdrl*:mCherry/*cmyb*:EGFP) embryo from 34 hpf to 40 hpf by Andor Dragonfly 505 confocal microscope. The white arrows indicate the HEC and emerging HSPCs.

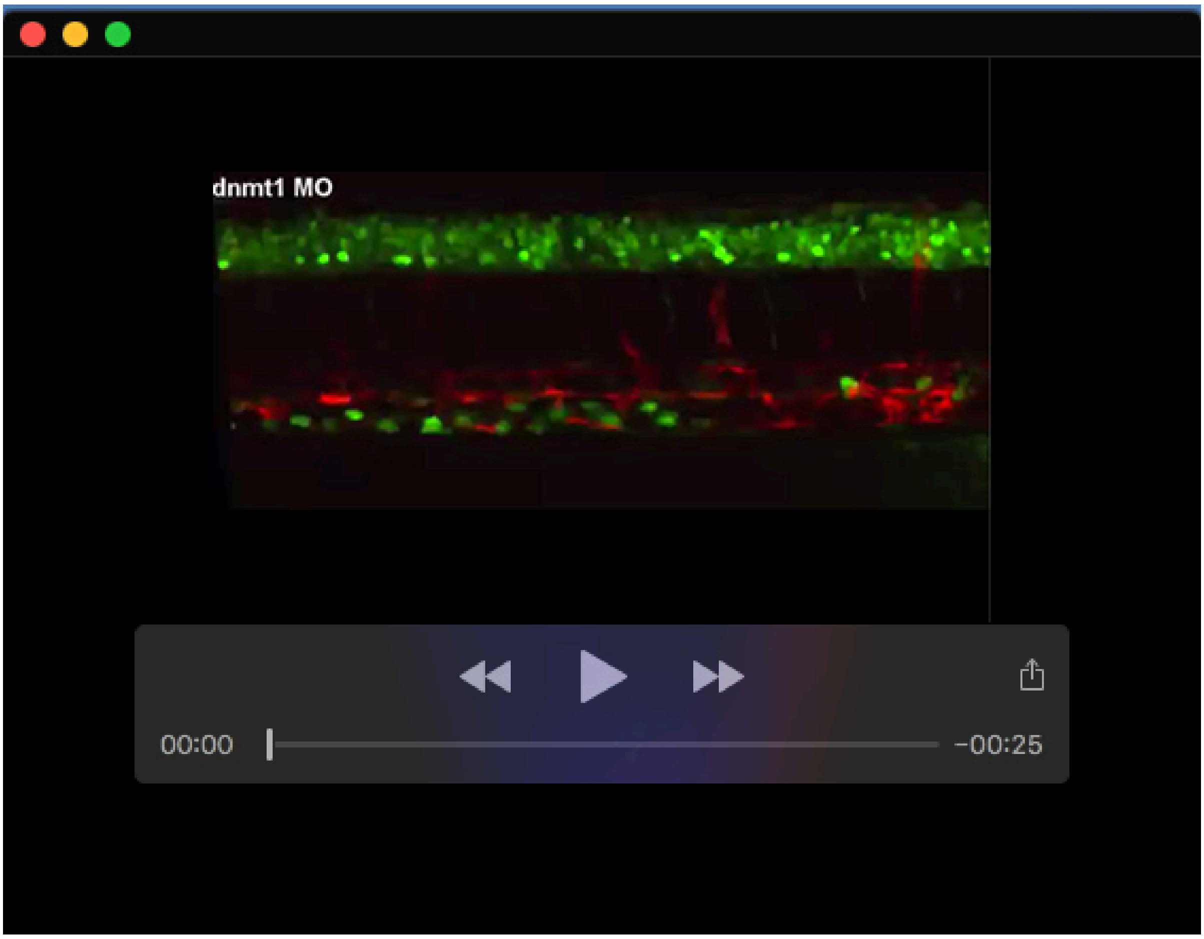

Movie 2. Time-lapse lineage tracing of EHT in *dnmt1* atgMO-injected embryos. The fate transition via EHT was impaired in *dnmt1* morphant Tg(*kdrl*:mCherry/*cmyb*:EGFP) embryo from 34 hpf to 40 hpf. The white arrow indicates the HEC and emerging HSPC. .

Development • Supplementary information
