# Supplementary material for: DNA methylation safeguards the generation of hematopoietic stem and progenitor cells by repression of Notch signaling
Source: Development. 2022 May 25;149(10):dev200390. doi: 10.1242/dev.200390 (PMC9188753; doi:10.1242/dev.200390)
Supplement: Reviewer comments [file develop-149-200390_review_history.pdf]

DNA methylation safeguards the generation of hematopoietic stem and progenitor cells by repression of Notch signaling

Yan Li, Chao Tang, Fan Liu, Caiying Zhu, Feng Liu, Ping Zhu and Lu WANG
DOI: 10.1242/dev.200390

Editor: Hanna Mikkola

Review timeline

Original submission:	24 November 2021
Editorial decision:	1 February 2022
First revision received:	24 March 2022
Accepted:	21 April 2022

Original submission

First decision letter

MS ID#: DEVELOP/2021/200390

MS TITLE: DNA methylation safeguards endothelial-to-hematopoietic transition by repression of Notch signaling

AUTHORS: Lu WANG, Yan Li, Chao Tang, Fan Liu, Caiying Zhu, Feng Liu, and Ping Zhu

I have now received all the referees' reports on the above manuscript, and have reached a decision. The referees' comments are appended below, or you can access them online: please go to BenchPress and click on the 'Manuscripts with Decisions' queue in the Author Area.

As you will see, the referees express considerable interest in your work, but have some significant criticisms and recommend a substantial revision of your manuscript before we can consider publication. If you are able to revise the manuscript along the lines suggested, which may involve further experiments, I will be happy receive a revised version of the manuscript. Your revised paper will be re-reviewed by one or more of the original referees, and acceptance of your manuscript will depend on your addressing satisfactorily the reviewers' major concerns. Please also note that Development will normally permit only one round of major revision.

We are aware that you may be experiencing disruption to the normal running of your lab that make experimental revisions challenging. If it would be helpful, we encourage you to contact us to discuss your revision in greater detail. Please send us a point-by-point response indicating where you are able to address concerns raised (either experimentally or by changes to the text) and where you will not be able to do so within the normal timeframe of a revision. We will then provide further guidance. Please also note that we are happy to extend revision timeframes as necessary.

Please attend to all of the reviewers' comments and ensure that you clearly highlight all changes made in the revised manuscript. Please avoid using 'Tracked changes' in Word files as these are lost in PDF conversion. I should be grateful if you would also provide a point-by-point response detailing how you have dealt with the points raised by the reviewers in the 'Response to Reviewers' box. If you do not agree with any of their criticisms or suggestions please explain clearly why this is so.

© 2022. Published by The Company of Biologists under the terms of the Creative Commons Attribution License (https://creativecommons.org/licenses/by/4.0/).

Reviewer 1

Advance summary and potential significance to field

In this manuscript, Li et al demonstrate a role for Dnmt1 in generation of HSCP from HE in the zebrafish model and connect its function to the attenuation of Notch signaling during EHT. Specifically, the authors first took an unbiased approach to evaluate the whole methylome of ECs, HECs and HSPCs sorted from zebrafish AGM by performing whole genome bisulfite sequencing, which demonstrated an HEC-specific hypermethylation pattern enriched in endothelial and Notch pathway genes. Having identified dnmt1 as an HSCP (cmyb+)-specific dnmt family member, the authors then showed dnmt1 deficiency resulted in reduction of HEC HSPC, and downstream progeny without affecting primitive hematopoiesis. Moreover they showed that the requirement for dnmt1 was restricted to the window of EHT and functioned through downregulation of endothelial-specific genes during EHT.
Next, comparing WGBS and RNA-seq of wt and dnmt1 ko EC, HEC, and HSCP, the authors identified specific deregulation of notch pathway genes in the setting of dnmt1 deficiency. Finally, they show a rescue of the HSCP defect in dnmt1 mutants by pharmacologic inhibition of Notch activity during the EHT window demonstrating a role for Notch inhibition downstream of dnmt1 as a major mechanism for promoting EHT.

Overall, the manuscript is well-written, the experiments are well designed, and the authors make use of complementary approaches throughout to validate their findings. The authors identify a novel mechanism for regulation of the Notch pathway during EHT in definitive hematopoiesis, adding to multiple other mechanisms that contribute to attenuation of Notch signaling which has been described to be essential for HSPC formation. While likely beyond the scope of the current paper, it would be interesting to know if the mechanism described in this manuscript extends to mammals (egs using mouse models or human PSC-derived definitive HEC) and how dnmt1 deficiency specifically affects functional HSCs vs HSC-independent lineages of LMPs, etc.

Comments for the author

Overall, I believe the manuscript is of suitable quality and significance for publication in Development, though I would recommend the authors address the following in a revised manuscript:
1) Throughout most of the manuscript, embryonic WISH staining is shown in representative samples but there is no quantitation of differences to enable a more robust statistical comparison. This is particularly important for some of the comparisons in which the differences are relatively subtle (egs. Fig 3F). For such comparisons, is there an unbiased way to quantify relative expression in the WISH studies (egs, using imaging software)? The results would be more convincing if relative expression was analyzed quantitatively in multiple independent experiments to show statistical significance of the findings.
2) For all experiment, the number of replicates/independent experiments should be stated in the figure legends (egs Fig 1b, 4c).
3) Line 47: HE is initially used as the abbreviation for hemogenic endothelium, but the manuscript subsequently switches to HEC. One abbreviation or the other should be used consistently throughout to avoid confusion.
4) Line 253-254: “which negatively regulates hematopoietic fate at the upstream of Notch signaling” should read “which negatively regulates hematopoietic fate upstream of Notch signaling”
5) For WISH figures, please describe in the legend what the arrowheads and the numbers in the lower right corner indicate.
6) Figure 4a: In mammalian hematopoiesis, some Notch receptors/ligands and target genes are specifically expressed in EC/HEC (egs. Dll4, Notch1, Hey1 Hey2), whereas others are more highly expressed in HSPC and/or required for their formation (egs Jag1, Notch2, Hes1). Can the authors speculate as to why there is general deregulation of all examined Notch receptors and target genes examined in Fig 4a? Were jagged ligands examined in this study? If so, was their expression also deregulated by Dnmt1 deficiency?
7) Figure 4f-g: Did the differences in DNA-methylation in dnmt1 mutant HEC extend to Notch target genes (hey2, etc), or is the higher expression of these genes in dnmt1 mutants (as shown in Fig 4a) purely a consequence of higher notch signaling activity?

© 2022. Published by The Company of Biologists under the terms of the Creative Commons Attribution License (https://creativecommons.org/licenses/by/4.0/).

8) Figure 5g: For those not familiar with bisulfite sequencing results, a clearer description in the legend of the how to interpret the results shown in the figure would be helpful.

Reviewer 2

Advance summary and potential significance to field

The authors performed whole genome bisulfite sequencing (WGBS) of zebrafish endothelial cells (ECs), hemogenic endothelial cells (HECs) and hematopoietic stem and progenitor cells (HSPCs) at 36hpf and identified the differentially methylated regions (DMRs) between these types of cells during development. They identified Dnmt1 as a potential player for HSPC development. Then they showed that in Dnmt1 mutant embryos, HSPC development was blocked and their derivatives, erythroid and lymphoid cells were impaired. They also used Dnmt1 translation-blocking morpholino and got similar phenotypes. Then they used Dnmt1 splicing-blocking morpholino and got similar phenotypes. Moreover, the authors performed rescue experiments by Dnmt1 mis-mRNAs and confirmed that HSPC development can be rescued by full-length Dnmt1 mis-mRNAs, but not by truncated Dnmt1 mis-mRNAs, suggesting that the methyltransferase activity of Dnmt1 is critical for HSPC development. Next the authors performed WGBS and RNA-seq of ECs, HECs, and HSPCs in Dnmt1 mutant and sibling embryos at 36hpf and identified the downstream target genes of Dnmt1. They showed that Notch signaling is abnormally upregulated in HECs in Dnmt1 mutants. They used a Notch inhibitor DBZ to treat Dnmt1 mutant Zebrafish embryos or Dnmt1 splicing-blocking morpholino injected Zebrafish embryos and showed that it can rescue HSPC development in both cases. The work is well designed and the data are solid and convincing. The work offers novel insights for the critical roles of DNA methylation for HSPC development through repressing Notch signaling pathway. However, it needs minor revisions:

1. The details of the sequencing data (WGBS and RNA sequencing) should be added in a supplementary table, such as sequencing depth, bisulfite conversion rate coverage, mapping rate.

2. Line 69: ‘but’ should be ‘and’ or something like that.

3. Line 198: ‘western’ should be ‘Western’.

4. Fig. 4G: The percentage of DNA methylation for each gene locus in mutant and control HECs should be shown.

Comments for the author

1. The details of the sequencing data (WGBS and RNA sequencing) should be added in a supplementary table, such as sequencing depth, bisulfite conversion rate coverage, mapping rate.
2. Line 69: ‘but’ should be ‘and’ or something like that.
3. Line 198: ‘western’ should be ‘Western’.
4. Fig. 4G: The percentage of DNA methylation for each gene locus in mutant and control HECs should be shown.

Reviewer 3

Advance summary and potential significance to field

In this paper, the authors tried to decipher the DNA methylation regulation of Notch signaling during endothelial-to-hematopoietic transition (EHT) in the zebrafish. They performed whole genome bisulfite sequencing of ECs, HECs and HSPCs, and identified DMRs that might be related to Notch signaling pathway.
Then, they knocked out / down dnmt1 to destroy DNA methylation maintenance and found that EHT was impaired in dnmt1 mutants and morphants, as revealed by altered expression levels of related marker genes, which could be rescued by overexpression of dnmt1 in morphants. In sum, by DNA methylation and transcriptome combined analysis and functional assays, the authors proposed that Dnmt1 is essential for EHT via repressing Notch signaling.

© 2022. Published by The Company of Biologists under the terms of the Creative Commons Attribution License (https://creativecommons.org/licenses/by/4.0/).

Comments for the author

In this paper, the authors tried to decipher the DNA methylation regulation of Notch signaling during endothelial-to-hematopoietic transition (EHT) in the zebrafish. They performed whole genome bisulfite sequencing of ECs, HECs and HSPCs, and identified DMRs that might be related to Notch signaling pathway. Then, they knocked out / down *dnmt1* to destroy DNA methylation maintenance and found that EHT was impaired in *dnmt1* mutants and morphants, as revealed by altered expression levels of related marker genes, which could be rescued by overexpression of *dnmt1* in morphants. In sum, by DNA methylation and transcriptome combined analysis and functional assays, the authors proposed that Dnmt1 is essential for EHT via repressing Notch signaling. However, the conclusion needs more compelling evidence.

Major concerns:
1, It remains obscure if dnmt1 functions during EHT in a cell-autonomous or cell non- autonomous fashion. It is important to investigate the dynamics of dnmt1 expression in ECs, HECs and HSPCs in the AGM region, not just in the CHT. The authors performed rescue experiment via *fli1a* promoter-driven transient expression of *dnmt1* in *dnmt1* morphants. Even if the rescue was real, it does not support the idea that dnmt1 directly functions in the HECs/HSPCs because fli1a also expresses in non-endothelia cells. Cell transplantation experiment may help address the question.

2, The rescue results with *dnmt1* overexpression were not convincing. In Fig. 2D, the number of *runx1*-positive cells should be counted and compared among groups, rather than a presentation of embryo proportions with different expression patterns. Similarly in Fig. 3H, the number of *cmyb*-positive cells should be counted and compared; importantly, the correlation of Dnmt1-EGFP expression signal and *cmyb* signal should be shown. Actually, rescue experiments should be performed in *dnmt1* mutants instead of morphants.

3, The model in Fig. 4H was neither explained in the text nor in the figure legend. It looks like Dnmt1 acts as *de novo* methyltransferase to silence notch genes. It is well known that Dnmt1 acts to maintain DNA methylation rather than de nova methylate DNA. As described in the Introduction section, Notch signaling is required for HEC specification and its repression is necessary for HSPCs emergence. Then, a de nova methylase such as Dnmt3a/b should function to methylate Notch genes following HEC fate specification and resulting DNA methylation is maintained by Dnmt1. Therefore, it is worthwhile investigating involvement of *dnmt3a/b* during EHT.

4, The authors mainly focused on the dynamics of several marker genes in *dnmt1* mutants or morphants, which is not sufficient to conclude that defective EHT is caused by loss of DNA methylation. The performed combinatory analysis of DNA methylation and transcriptome is less informative.

5, What is the exact DMRs distribution in genome in sFig.1B? Numbers or ratios of different regions might be more informative than odds Ratio. And GO analysis of DMRs in Fig. 1C and sFig. 1D should be replaced with GREAT analysis, which would be more reliable, due to the authors presented that most DMRs are not located in promoter regions in sFig.1B.

6, It is necessary to present the distribution of DMRs in mutants and siblings (related with sFig. 5A), because it is a precondition for subsequent combinatory analysis of promoter DNA hypomethylation and DEGs.

7, Besides related marker genes they checked by qPCR, how many total DEGs were present in *dnmt1* mutants, and what are they? For negative correlation analysis, the authors should add more details in the Methods section, and give some examples of genes showing both RNA level and promoter DNA methylation level.

8, For other key factors that regulate silencing of Notch signaling pathway, such as *gpr183*, *blos2*, miR233 and so on, what are DNA methylation states of these genes in WT and mutant?

9, Is it possible to overexpress *tet* genes driven by HSP70 or *fila* promoters, to validate relationship between DNA methylation and Notch gene expression?

© 2022. Published by The Company of Biologists under the terms of the Creative Commons Attribution License (https://creativecommons.org/licenses/by/4.0/).

Minor points:
10, Fig. 3H lacks caption.

11, In Fig. 4F, the DMRs should be zoomed in to make them clearer.

12, In Fig. 4G, the regions of PCR products should be labeled.

13, In western blot results, it would be better to add marker and stage information.

First revision

Author response to reviewers' comments

Response to the reviewers' comments

We are very grateful to the reviewers for their insightful comments on our manuscript. We have performed a number of critical experiments and analysis suggested to improve the quality of our manuscript, including transplantation experiments and rescue experiments in *dnmt1* mutant to validate the phenotypes. The detailed point-by-point responses to reviewers' comments are shown below.

Reviewer 1 Advance Summary and Potential Significance to Field:
In this manuscript, Li et al demonstrate a role for Dnmt1 in generation of HSCP from HE in the zebrafish model and connect its function to the attenuation of Notch signaling during EHT. Specifically, the authors first took an unbiased approach to evaluate the whole methylome of ECs, HECs and HSPCs sorted from zebrafish AGM by performing whole genome bisulfite sequencing, which demonstrated an HEC-specific hypermethylation pattern enriched in endothelial and Notch pathway genes. Having identified dnmt1 as an HSCP (cmyb+)-specific dnmt family member, the authors then showed dnmt1 deficiency resulted in reduction of HEC, HSPC, and downstream progeny without affecting primitive hematopoiesis. Moreover, they showed that the requirement for dnmt1 was restricted to the window of EHT and functioned through downregulation of endothelial-specific genes during EHT. Next, comparing WGBS and RNA-seq of wt and dnmt1 ko EC, HEC, and HSCP, the authors identified specific deregulation of notch pathway genes in the setting of dnmt1 deficiency. Finally, they show a rescue of the HSCP defect in dnmt1 mutants by pharmacologic inhibition of Notch activity during the EHT window, demonstrating a role for Notch inhibition downstream of dnmt1 as a major mechanism for promoting EHT.

Overall, the manuscript is well-written, the experiments are well designed, and the authors make use of complementary approaches throughout to validate their findings. The authors identify a novel mechanism for regulation of the Notch pathway during EHT in definitive hematopoiesis, adding to multiple other mechanisms that contribute to attenuation of Notch signaling which has been described to be essential for HSPC formation.
While likely beyond the scope of the current paper, it would be interesting to know if the mechanism described in this manuscript extends to mammals (egs using mouse models or human PSC-derived definitive HEC)

We thank the reviewer for the positive and encouraging comments. A recent study described a DNA methylation landscape during mouse HSC development, and revealed that the endothelial-featured genes undergo gain-of-methylation in T1 pre-HSCs (Li et al., 2021). Although there was a lack of experimental investigations, their findings were consistent with the mechanisms in our manuscript.
and how dnmt1 deficiency specifically affects functional HSCs vs HSC-independent lineages of LMPs, etc.

Based on our results showing unaffected HSC-independent erythroid and myeloid lineages and impaired HSPCs upon *dnmt1* deficiency, we speculate that the reason underlying these differences might be the specific expression of *dnmt1* in HSPC/HEC as we showed in Figure S2A; alternatively,

© 2022. Published by The Company of Biologists under the terms of the Creative Commons Attribution License (https://creativecommons.org/licenses/by/4.0/).

there might be different tolerance for alteration of DNA methylation in HSCs and HSC-independent lineages.

Reviewer 1 Comments for the Author:
Overall, I believe the manuscript is of suitable quality and significance for publication in Development, though I would recommend the authors address the following in a revised manuscript:
1) Throughout most of the manuscript, embryonic WISH staining is shown in representative samples but there is no quantitation of differences to enable a more robust statistical comparison. This is particularly important for some of the comparisons in which the differences are relatively subtle (egs. Fig 3F). For such comparisons, is there an unbiased way to quantify relative expression in the WISH studies (egs, using imaging software)? The results would be more convincing if relative expression was analyzed quantitatively in multiple independent experiments to show statistical significance of the findings.

Response 1. Thanks for this suggestion. We have performed quantification using ImageJ and statistical analysis of the WISH results (Dobrzycki et al., 2020) , and then have added the analysis in the revised Figures (see revised Fig 2B, 2F; Fig 4B, 4E; Fig 5E; Fig S2C, 2E, 2I; Fig S3C, 3F, 3H; Fig S4B, 4F, 4I; Fig S5F, S5H; Fig S6C).

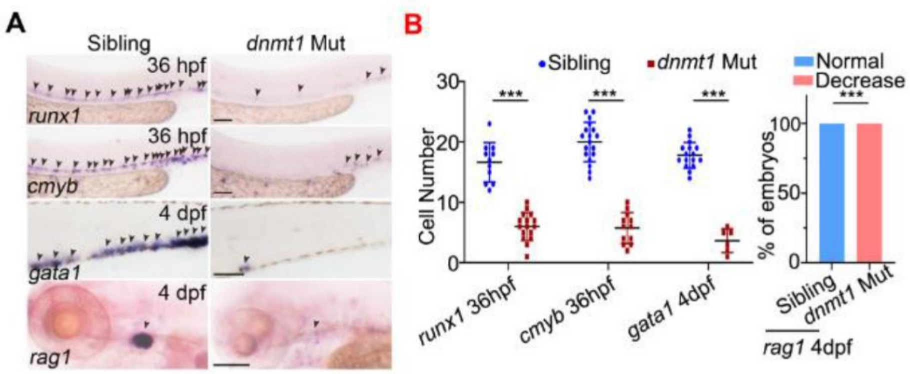

2) For all experiment, the number of replicates/independent experiments should be stated in the figure legends (egs Fig 1b, 4c).

Response 2. We have added the description in the revised figure legend as it writes “n≥3 replicates.” now.

3) Line 47: HE is initially used as the abbreviation for hemogenic endothelium, but the manuscript subsequently switches to HEC. One abbreviation or the other should be used consistently throughout to avoid confusion.

Response 3. Thanks for pointing out this. We have revised and used “HEC” in revised the manuscript.

4) Line 253-254: “which negatively regulates hematopoietic fate at the upstream of Notch signaling” should read “which negatively regulates hematopoietic fate upstream of Notch signaling”

Response 4. Thanks for the suggestion. We have revised the manuscript and deleted this sentence.

5) For WISH figures, please describe in the legend what the arrowheads and the numbers in the lower right corner indicate.

Response 5. Thanks for pointing out this. We have added the description of arrowheads in WISH figures as it writes “The arrowheads indicate the expression of HSPC marker *runx1* (revised Fig2E)”. The numbers below the WISH figures indicate the number of embryos showing

© 2022. Published by The Company of Biologists under the terms of the Creative Commons Attribution License (https://creativecommons.org/licenses/by/4.0/).

representative phenotype/total number of embryos analyzed in each experiment. We have revised the figures and performed quantification and statistical analysis of WISH results.

6) Figure 4a: In mammalian hematopoiesis, some Notch receptors/ligands and target genes are specifically expressed in EC/HEC (egs. Dll4, Notch1, Hey1, Hey2), whereas others are more highly expressed in HSPC and/or required for their formation (egs Jag1, Notch2, Hes1). Can the authors speculate as to why there is general deregulation of all examined Notch receptors and target genes examined in Fig 4a? Were jagged ligands examined in this study? If so, was their expression also deregulated by Dnmt1 deficiency?

Response 6. Based on the present and previous findings, Notch signaling pathway plays distinct roles during EHT, including HEC specification and HSPC formation, through different receptors/ligands. Notch signaling should be precisely controlled and we speculate that DNA methylation fine-tunes the expression of receptors and ligands. Besides the results showed that the methylation and expression of Notch receptors were affected upon *dnmt1* deficiency, we also demonstrated that the methylation level of jagged ligands (*jag1a* and *jag1b*) was also decreased and correspondingly, the expression level of *jag1a* was increased in *dnmt1* mutant HEC compared with siblings (see below **Response Figure 1**). The methylation level of most Notch target genes was unaffected by *dnmt1* deficiency, but the expression level was upregulated (see below **Response Figure 1** and **Fig. 4A**). We speculate that the increased expression of target genes was attributed to higher Notch signaling activity upon *dnmt1* deficiency.

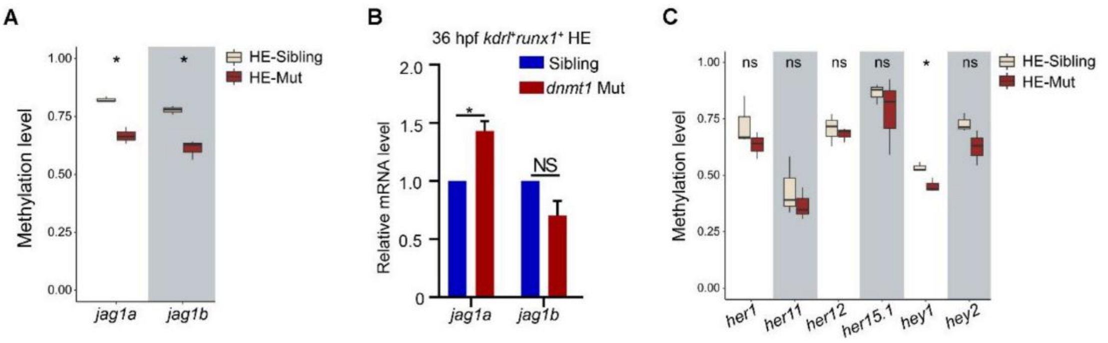

Response Figure 1. The methylation level and transcription level of Notch ligands and target genes.
(A) Analysis of WGBS data showing the decreased methylation level of *jag1a* and *jag1b* in *dnmt1* mutant.
(B) q-PCR results showing the expression of *jag1a* and *jag1b*. (C) Analysis of WGBS data showed that the methylation level of most Notch target genes was unaffected by *dnmt1* deficiency.

7) Figure 4f-g: Did the differences in DNA-methylation in dnmt1 mutant HEC extend to Notch target genes (hey2, etc), or is the higher expression of these genes in dnmt1 mutants (as shown in Fig 4a) purely a consequence of higher notch signaling activity?

Response 7. Thanks for pointing out this. As mentioned above in Response 6, we have shown that the methylation level of target genes was unaffected upon *dnmt1* deficiency (see **Response Figure 1C)**. Therefore, we speculate that the higher expression of these genes in *dnmt1* mutants is purely a consequence of higher Notch signaling activity.

8) Figure 5g: For those not familiar with bisulfite sequencing results, a clearer description in the legend of the how to interpret the results shown in the figure would be helpful.

Response 8. Thank you for your suggestion. We have revised the legends to describe the bisulfite sequencing results as “Bisulfite sequencing analysis of DNA methylation at DMRs in *notch1a*, *notch1b*, *notch* and *notch3* in sibling-HEC and *dnmt1* mutant-HEC. Filled circle, methylated CpG. Unfilled circle, unmethylated CpG. The number underneath each sequencing diagram indicates the percentage of all methylated CpG sites over the total number of CpG sites of the sequenced colonies for each sample.”

© 2022. Published by The Company of Biologists under the terms of the Creative Commons Attribution License (https://creativecommons.org/licenses/by/4.0/).

Reviewer 2 Advance Summary and Potential Significance to Field:
The authors performed whole genome bisulfite sequencing (WGBS) of zebrafish endothelial cells (ECs), hemogenic endothelial cells (HECs) and hematopoietic stem and progenitor cells (HSPCs) at 36hpf and identified the differentially methylated regions (DMRs) between these types of cells during development. They identified Dnmt1 as a potential player for HSPC development. Then they showed that in Dnmt1 mutant embryos, HSPC development was blocked and their derivatives, erythroid and lymphoid cells were impaired. They also used Dnmt1 translation-blocking morpholino and got similar phenotypes. Then they used Dnmt1 splicing-blocking morpholino and got similar phenotypes. Moreover, the authors performed rescue experiments by Dnmt1 mis-mRNAs and confirmed that HSPC development can be rescued by full-length Dnmt1 mis-mRNAs, but not by truncated Dnmt1 mis-mRNAs, suggesting that the methyltransferase activity of Dnmt1 is critical for HSPC development. Next the authors performed WGBS and RNA-seq of ECs, HECs, and HSPCs in Dnmt1 mutant and sibling embryos at 36hpf and identified the downstream target genes of Dnmt1. They showed that Notch signaling is abnormally upregulated in HECs in Dnmt1 mutants. They used a Notch inhibitor DBZ to treat Dnmt1 mutant Zebrafish embryos or Dnmt1 splicing-blocking morpholino injected Zebrafish embryos and showed that it can rescue HSPC development in both cases. The work is well designed and the data are solid and convincing. The work offers novel insights for the critical roles of DNA methylation for HSPC development through repressing Notch signaling pathway.
However, it needs minor revisions:

1. The details of the sequencing data (WGBS and RNA sequencing) should be added in a supplementary table, such as sequencing depth, bisulfite conversion rate, coverage, mapping rate.

Response 9. Thanks for your suggestion. We have added the details of sequencing data in revised Tables S3, S4, S5 and S6.

2. Line 69: 'but' should be 'and' or something like that.

Response 10. We have revised the manuscript (Line 71).

3. Line 198: 'western' should be 'Western'.

Response 11. We have revised the manuscript (Line 203).

4. Fig. 4G: The percentage of DNA methylation for each gene locus in mutant and control HECs should be shown.

Response 12. Thanks for this suggestion. We have added the percentage of DNA methylation level in revised Figure 6B.

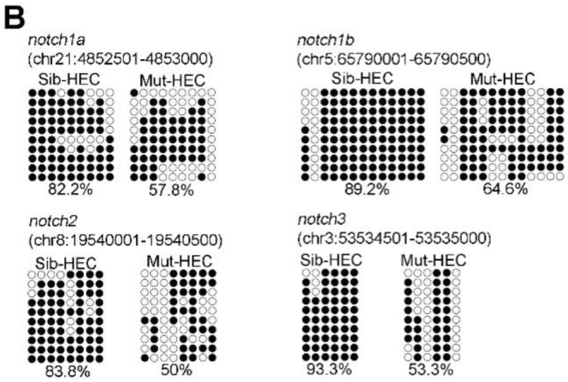

Reviewer 3
In this paper, the authors tried to decipher the DNA methylation regulation of Notch signaling during endothelial-to-hematopoietic transition (EHT) in the zebrafish. They performed whole genome bisulfite sequencing of ECs, HECs and HSPCs, and identified DMRs that might be

© 2022. Published by The Company of Biologists under the terms of the Creative Commons Attribution License (https://creativecommons.org/licenses/by/4.0/).

related to Notch signaling pathway. Then, they knocked out / down dnmt1 to destroy DNA methylation maintenance and found that EHT was impaired in dnmt1 mutants and morphants, as revealed by altered expression levels of related marker genes, which could be rescued by overexpression of dnmt1 in morphants. In sum, by DNA methylation and transcriptome combined analysis and functional assays, the authors proposed that Dnmt1 is essential for EHT via repressing Notch signaling.
However, the conclusion needs more compelling evidence.

Major concerns:
1, It remains obscure if dnmt1 functions during EHT in a cell-autonomous or cell nonautonomous fashion. It is important to investigate the dynamics of dnmt1 expression in ECs, HECs and HSPCs in the AGM region, not just in the CHT.

Response 13. We thank this reviewer for pointing out this important issue. We have examined *dnmt1* expression at 36 hpf in the AGM region using double fluorescence in situ hybridization (**Response Figure 2A** and revised **Fig. S2A**). The results showed that the expression of *dnmt1* was enriched in *cmyb/runx1*$^{+}$ cells (including HECs and HSPCs), while only some of *fli1a*$^{+}$ ECs displayed high and specific *dnmt1* expression. And we also have examined expression of *dnmt1* using our RNA-seq data in EC, HEC and HSPC. The results showed that the expression of *dnmt1* in HECs and HSPCs was obviously higher than that in ECs (**Response Figure 2B).**

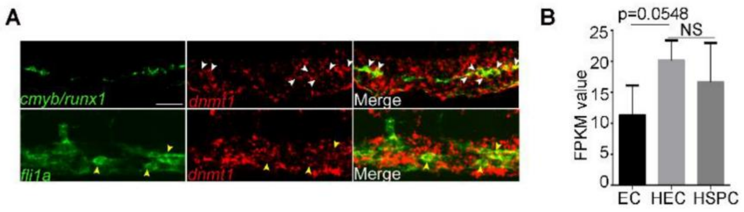

Response Figure 2. The expression of *dnmt1*. (A) Double fluorescence in situ hybridization (FISH) analysis showing co-expression of *dnmt1* with *cmyb/runx1* (white arrowheads) and *dnmt1* with *fli1a* (yellow arrowheads) in the AGM region at 36 hpf, respectively. Scale bars, 30 μm. (B) FPKM value of *dnmt1* expression in RNA-seq data.

The authors performed rescue experiment via fli1a promoter-driven transient expression of dnmt1 in dnmt1 morphants. Even if the rescue was real, it does not support the idea that dnmt1 directly functions in the HECs/HSPCs because fli1a also expresses in non-endothelia cells. Cell transplantation experiment may help address the question.

This is a critical point. To determine whether Dnmt1 is required cell autonomously for HSPC generation, we have performed blastula transplant experiment. Donor cells labeled by rhodamine from *cmyb*:EGFP embryos were transplanted at the blastula stage into nontransgenic recipients. The results showed that 4/10 wild type recipient embryos and 6/15 *dnmt1* morphant recipients had a few GFP^{+} HSPCs derived from wild type donor cells, whereas only few wild type recipient embryos (2/41) had GFP^{+} HSPCs from donor cells of *dnmt1* morphants (**Response Figure 3A, B and revised Fig. 2H, 2I**). Together, the transplantation results suggested that blastula cells lacking *dnmt1* hardly gave rise to *cmyb*$^{+}$ HSPCs in normal recipients.

Besides, to further investigate the direct role of *dnmt1* in HECs/HSPCs, we have performed HSPC- specific *dnmt1* overexpression driven by a mouse *runx1*+24 enhancer and Hbb minimal promoter (to ensure minimal activity). The results showed that *dnmt1* overexpression in HSPCs efficiently rescued the decrease of HSPCs in AGM region in *dnmt1* morphants (**Response Figure 3C, D**).
Taken together, these data suggested that Dnmt1 is required for HSPC generation cell autonomously.

© 2022. Published by The Company of Biologists under the terms of the Creative Commons Attribution License (https://creativecommons.org/licenses/by/4.0/).

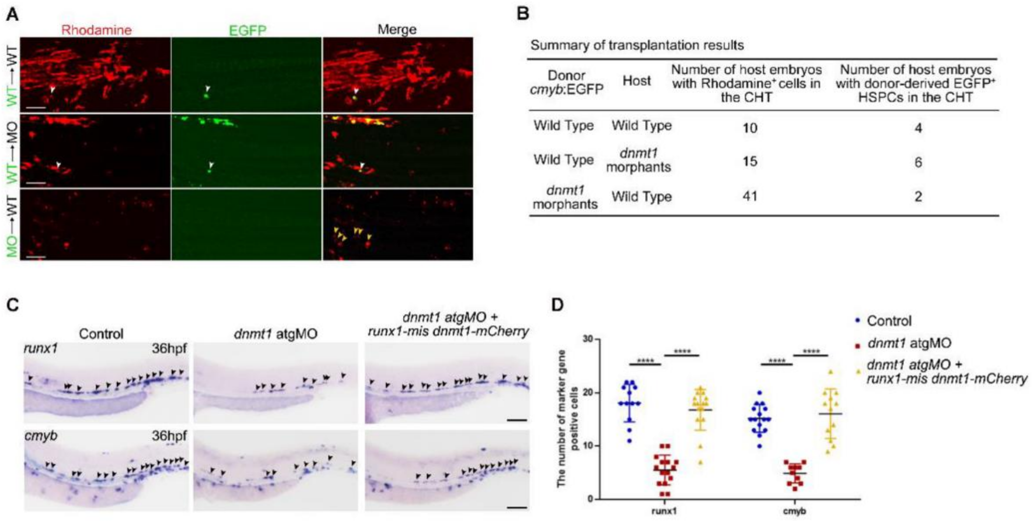

Response Figure 3. Dnmt1 is required HSPC generation cell autonomously. (A) and (B) Transplantation results showing HSPC reconstitution in the CHT region of recipient embryos at 36 hpf. Green, *cmyb*$^{+}$ EGFP cells; red, rhodamine; white arrowheads, EGFP^{+} HSPCs contributed by donor cells; yellow arrowheads, donor-derived EGFP^{-} hematopoietic cells in CHT region. CHT, caudal hematopoietic tissue. (C) Expression of *runx1* and *cmyb* in control, *dnmt1* morphants, and embryos-coinjected with *dnmt1* atgMO and *runx1*: mismatch-*dnmt1*-mCherry constructs. Scale bar, 100 μm. The arrowheads indicate the expression of *runx1* and *cmyb*. n≥3 replicates. (D) Statistical analysis of the WISH data in (C). Error bars, mean ± SD, ****P < 0.0001, student's t test.

2, The rescue results with dnmt1 overexpression were not convincing. In Fig. 2D, the number of runx1- positive cells should be counted and compared among groups, rather than a presentation of embryo proportions with different expression patterns. Similarly in Fig. 3H, the number of cmyb-positive cells should be counted and compared;

Response 14. Thanks for this suggestion. We have performed quantification and statistical analysis of all WISH results using imageJ, then we have added the statistical analysis in the revised Figures, including *dnmt1* overexpression experiments (see revised Fig 2F; Fig 4D, 4E; Fig 5E; Fig S4F, 4I; Fig S5H; Fig S6C).

importantly, the correlation of Dnmt1-EGFP expression signal and cmyb signal should be shown.

This is a critical point. To examine the correlation of Dnmt1-EGFP expression signal and *cmyb* expression, we performed double fluorescent in situ hybridization (FISH) to detect the expression of *cmyb* and *egfp*. The result showed that *cmyb* and *egfp* double positive cells were detected in the AGM region in *fli1a*-mis-*dnmt1*-EGFP-injected *dnmt1* morphants. We also found that the endothelial-derived Dnmt1-EGFP overexpression rescued the decreased population of *cmyb*$^{+}$ cells in the AGM region (**revised Fig. 4D**).

Actually, rescue experiments should be performed in dnmt1 mutants instead of morphants.

Thanks for this suggestion. We now have performed rescue experiments in *dnmt1* mutant, including *fli1a* promoter-mis Dnmt1 and *hsp70* promoter-mis Dnmt1 overexpression, respectively (**see revised Fig. 2E, 2F; Fig. 4D, 4E**). And the HSPC defects in *dnmt1* mutant were efficiently rescued by *hsp70* promoter- driven Dnmt1 overexpression (heated from 24 hpf) and *fli1a* promoter-driven Dnmt1 overexpression, respectively.

3, The model in Fig. 4H was neither explained in the text nor in the figure legend. It looks like Dnmt1 acts as de novo methyltransferase to silence notch genes. It is well known that Dnmt1 acts to maintain DNA methylation rather than de nova methylate DNA. As described in the Introduction section, Notch signaling is required for HEC specification and its repression is necessary for HSPCs

© 2022. Published by The Company of Biologists under the terms of the Creative Commons Attribution License (https://creativecommons.org/licenses/by/4.0/).

emergence. Then, a de nova methylase such as Dnmt3a/b should function to methylate Notch genes following HEC fate specification and resulting DNA methylation is maintained by Dnmt1. Therefore, it is worthwhile investigating involvement of dnmt3a/b during EHT.

Response 15. We apologize that we didn't fully describe the model in the original version. In this work, we have demonstrated that Dnmt1 could regulate Notch genes by modulating DNA methylation of these genes. However, we could not exclude the involvement of Dnmt3a/3b during HSPC emergence. Previous study has demonstrated that deficiency of *dnmt3bb.1* (previously known as dnmt4, the closest zebrafish ortholog of the mammalian DNMT3B) did not affect HSPC specification, but regulated maintenance of hematopoietic cell fate. And they also have detected the decreased methylation level and transcription level of *notch1b* upon *dnmt3bb.1* deficiency(Gore et al., 2016). So, we speculated that Dnmt3bb.1 may be involved in the regulation of DNA methylation of *notch1b*, but this modulation was not sufficient to affect HSPC specification in *dnmt3bb.1*-deficient embryos. We also have performed knockdown of *dnmt3ab* (previously known as *dnmt6*, the closest zebrafish ortholog of the mammalian DNMT3A), and then detected the HSPC phenotype and methylation level. The results showed that the expression of HSPC markers *runx1* and *cmyb* and artery markers (*dll4*, *dltC*, *ephrinB2* and *hey2*) was comparable with that in control embryos at 36 hpf and that the methylation level of Notch genes was unaffected (**Response Figure 4**). Collectively, Dnmt3ab and Dnmt3bb.1 were not involved in modulating methylation of Notch genes during HSPC generation.

And we have redrawn a new model to depict the role of Dnmt1-mediated methylation in regulation of HSPC generation through repression of Notch genes (**revised Fig. S6D and Response Figure 4F**). During normal development, Notch signaling is controlled by DNA methylation to ensure HSPC production. In the absence of *dnmt1*, Notch signaling is abnormally activated due to the hypomethylation, thereby causing impaired HSPC generation.

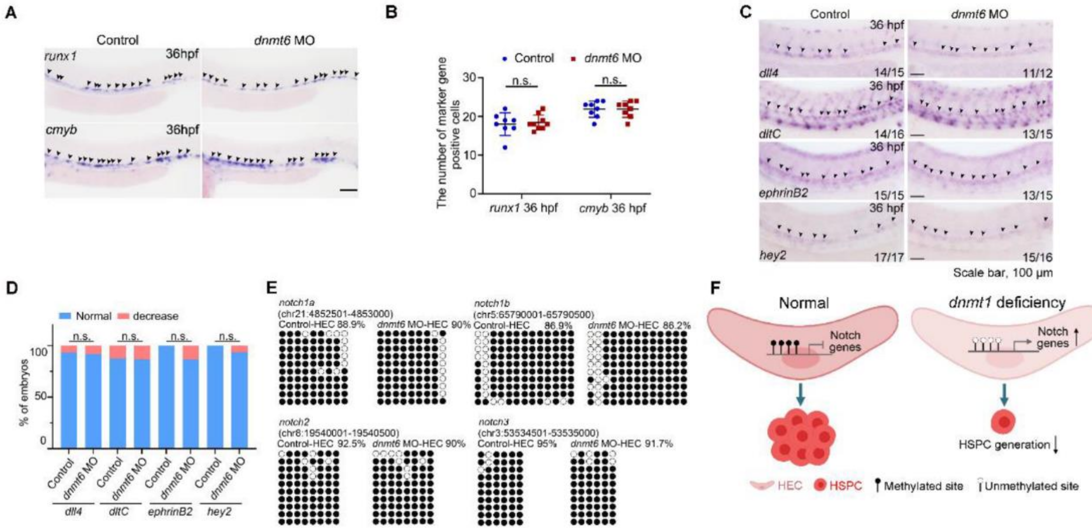

Response Figure 4. Loss of *dnmt6* did not affect the HSPC generation, artery formation and methylation of Notch genes. (A) Expression of *runx1* and *cmyb* in the AGM region at 36 hpf in control and *dnmt6*-deficient embryos. Scale bar, 100 μm. The arrowheads indicate the expression of *runx1* and *cmyb*. n≥3 replicates. (B) Statistical analysis of the WISH data in (A). Error bars, mean ± SD, NS, no significance, student's t test. (C) WISH analysis showing the expression of arterial endothelial genes *dll4*, *dltC*, *ephrinB2* and *hey2* in siblings and *dnmt1* mutants at 36 hpf. Scale bar, 100 μm. The arrowheads indicate the expression of corresponding arterial endothelial genes. n≥3 replicates. (D) Statistical analysis of the WISH in (C). Error bars, mean ± SD, n.s., no significance, student's t test. (E) Bisulfite sequencing analysis of DNA methylation at DMRs in *notch1a*, *notch1b*, *notch2* and *notch3* in control-HEC and *dnmt6* morphants-HEC. Filled circle, methylated CpG. Unfilled circle, unmethylated CpG. The number upon each sequencing diagram indicates the percentage of all methylated CpG sites over the total number of CpG sites of the sequenced colonies for each sample. (F) Model depicting the role of Dnmt1-mediated methylation in regulation of HSPC generation through repression of Notch genes.

© 2022. Published by The Company of Biologists under the terms of the Creative Commons Attribution License (https://creativecommons.org/licenses/by/4.0/).

4, The authors mainly focused on the dynamics of several marker genes in dnmt1 mutants or morphants, which is not sufficient to conclude that defective EHT is caused by loss of DNA methylation. The performed combinatory analysis of DNA methylation and transcriptome is less informative.

Response 16. Thanks for this suggestion.

1. We have analyzed RNAseq data and performed volcano plot and GO analysis. And GO analysis showed that the decreased genes in *dnmt1*-deficient HSPCs were involved in stem cell differentiation, hematopoietic stem cell differentiation and cell fate determination. Many notch genes were upregulated in *dnmt1* mutant, compared with siblings (**revised Fig. S5C and Fig 3C).** These results indicate the impaired HSPC emergence upon *dnmt1* deficiency.

2. We also have performed time-lapse imaging in control and *dnmt1*-deficient embryos to examine whether EHT process was impaired upon *dnmt1* deficiency. And the results confirmed that, compared to control with the fate transition occurring, ECs in *dnmt1*-deficient embryos failed to transit into HSPCs (**revised Movies S1 and S2**).

3. The combinatory analysis of WGBS and RNA-seq was to identify the direct regulation of transcriptome by methylation. Since DNA methylation usually acts as a repressive regulator of gene expression (Jones, 2012), we identified genes with negative correlation and further analyzed the genes with upregulated promoter methylation and decreased RNA expression in sibling samples, but not in *dnmt1* mutant. Negative correlations analysis showed that significant enrichment in vasculature development could be detected in sibling but not in mutant, indicating that *dnmt1* deficiency could destroy regulation between methylation and transcriptome. And our data showed that there was no significant enrichment in terms of hematopoietic development. We also have examined the methylation level of *runx1*, *gata2b* and *gfi1aa*, which are essential for HSPC generation, and found the methylation level of these genes was unaffected. These results indicate that Dnmt1 could not directly regulate hematopoietic genes by modulating DNA methylation (**Response Figure 5A, B and revised Fig 3D**). Collectively, DNA methylation specifically regulates endothelial-related genes to affect HSPC generation.

Taken together, our WISH data, and newly added EHT movie and RNA seq analysis together demonstrated that EHT process was affected in *dnmt1*-deficient embryos.

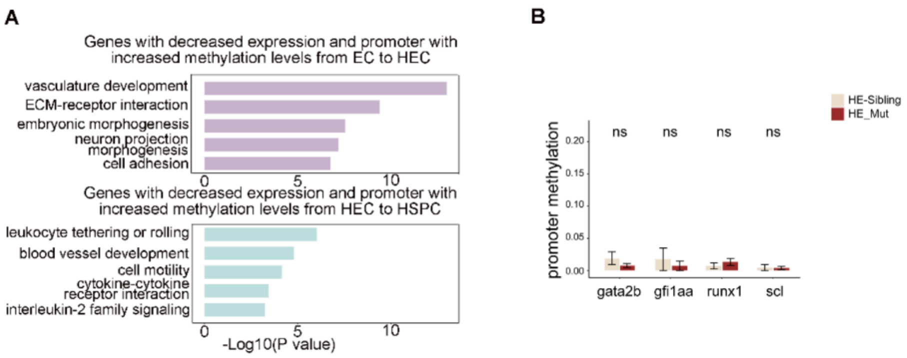

Response Figure 5. DNA methylation specifically regulates blood vessel-related genes but not hematopoietic genes. (A) Gene Ontology analysis of the genes inactivated during HSPC generation, while the methylation levels of their promoters increased. (B) Promoter methylation level of hematopoietic genes. Error bars, mean ± SD, NS, no significance, student's t test.

5, What is the exact DMRs distribution in genome in sFig.1B? Numbers or ratios of different regions might be more informative than odds Ratio. And GO analysis of DMRs in Fig. 1C and sFig. 1D should be replaced with GREAT analysis, which would be more reliable, due to the authors presented that most DMRs are not located in promoter regions in sFig.1B.

Response 17. Thanks for this suggestion. We have displayed DMR distribution in revised Fig S1B. And we also have changed GO analysis to GREAT analysis in revised Fig 1C and revised Fig 1E.

© 2022. Published by The Company of Biologists under the terms of the Creative Commons Attribution License (https://creativecommons.org/licenses/by/4.0/).

6, It is necessary to present the distribution of DMRs in mutants and siblings (related with sFig. 5A), because it is a precondition for subsequent combinatory analysis of promoter DNA hypomethylation and DEGs.

Response 18. Thanks for this suggestion. We have added DMR distribution in revised sFig 5B.

7, Besides related marker genes they checked by qPCR, how many total DEGs were present in dnmt1 mutants, and what are they? For negative correlation analysis, the authors should add more details in the Methods section, and give some examples of genes showing both RNA level and promoter DNA methylation level.

Response 19. Thanks for this suggestion. We have analyzed RNA-seq data and performed volcano plot and GO analysis. We found that 901 and 795 genes were upregulated and downregulated in *dnmt1* mutant, respectively. Intriguingly, genes with upregulated expression showed enrichment in blood vessel development, angiogenesis and artery development, while genes with decreased expression were enriched for terms associated with stem cell differentiation and hematopoietic progenitor cell differentiation. And we have added the analysis in revised manuscript (Revised Fig S5C and Fig 3C). And we have added details in correlation analysis into the revised method (Line 259-272), and displayed methylation and transcription level of many genes as samples in revised Table S7.

8, For other key factors that regulate silencing of Notch signaling pathway, such as gpr183, blos2, miR233 and so on, what are DNA methylation states of these genes in WT and mutant?

Response 20. We have examined the methylation level of *gpr183* and *blos2* and found that the methylation level of *blocs2* was unaffected, but *gpr183* displayed decreased methylation. We haven't detected *mir233* because of the depth of WGBS. Whether these previous reported Notch regulators are involved in DNA methylation-mediated HSPC emergence await further investigation.

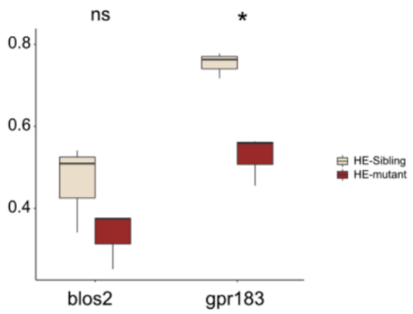

9, Is it possible to overexpress tet genes driven by HSP70 or fila promoters, to validate relationship between DNA methylation and Notch gene expression?

Response 21. The zebrafish genome encodes single orthologs of Tet1, Tet2, and Tet3. Previous study identified that *tet2* and *tet3* were the major 5mC dioxygenase and had overlapping roles in regulating hematopoiesis. The authors generated double-homozygous mutants and found that loss of *tet2*/*tet3* could decrease Notch signaling, thereby compromising HSC emergence (Li et al., 2015) We have generated HSP70-*tet2*-EGFP and HSP70-*tet3*-EGFP to overexpress *tet2* and *tet3* at HSPC generation stage respectively (from 24 hpf) and performed bisulfite-PCR to detect methylation level of Notch genes. The results showed that not all notch genes' methylation level could be affected upon *tet2*- and *tet3* overexpression. Only *notch1b* and *notch2* showed decreased methylation level, while other Notch genes displayed unaltered methylation. Based on this study and our findings in this manuscript, we speculate that DNA methylation level is essential for Notch signaling during HSC emergence.

© 2022. Published by The Company of Biologists under the terms of the Creative Commons Attribution License (https://creativecommons.org/licenses/by/4.0/).

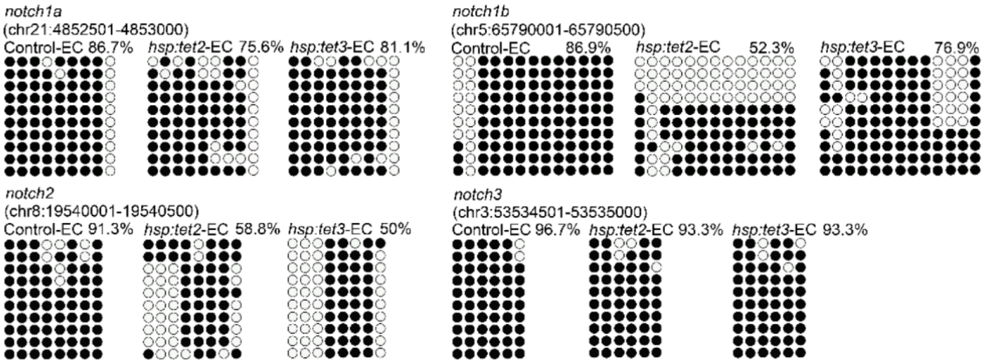

Minor points:
10, Fig. 3H lacks caption.

Response 22. We apologize for this error and we have added the caption in the revised Fig S5G.

11, In Fig. 4F, the DMRs should be zoomed in to make them clearer.

Response 23. We have added zoomed DMRs in revised Fig6A.

12, In Fig. 4G, the regions of PCR products should be labeled.

Response 24. We have added regions of bisulfite-PCR products in revised Fig 6B.

13, In western blot results, it would be better to add marker and stage information

Response 25. We have added the weight of each protein in revised Figures (Revised Fig 5B; Fig S2B; Fig S3A; Fig S4D and Fig S6A) and have added the stage information in legend, respectively.

References

Dobrzycki, T., Krecsmarik, M., and Monteiro, R. (2020). Genotyping and Quantification of In Situ Hybridization Staining in Zebrafish. Journal of visualized experiments : JoVE.
Gore, A.V., Athans, B., Iben, J.R., Johnson, K., Russanova, V., Castranova, D., Pham, V.N., Butler, M.G., Williams-Simons, L., Nichols, J.T., *et al.* (2016). Epigenetic regulation of hematopoiesis by DNA methylation. Elife 5, e11813.
Jones, P.A. (2012). Functions of DNA methylation: islands, start sites, gene bodies and beyond. Nat Rev Genet *13*, 484-492.
Li, C., Lan, Y., Schwartz-Orbach, L., Korol, E., Tahiliani, M., Evans, T., and Goll, M.G. (2015). Overlapping Requirements for Tet2 and Tet3 in Normal Development and Hematopoietic Stem Cell Emergence. Cell Rep *12*, 1133-1143.
Li, X., Liu, D., Zhang, L., Wang, H., Li, Y., Li, Z., He, A., Liu, B., Zhou, J., Tang, F., *et al.* (2021). The comprehensive DNA methylation landscape of hematopoietic stem cell development. Cell Discov *7*, 86.

© 2022. Published by The Company of Biologists under the terms of the Creative Commons Attribution License (https://creativecommons.org/licenses/by/4.0/).

Second decision letter

MS ID#: DEVELOP/2021/200390

MS TITLE: DNA methylation safeguards the generation of hematopoietic stem and progenitor cells by repression of Notch signaling

AUTHORS: Yan Li, Chao Tang, Fan Liu, Caiying Zhu, Feng Liu, Ping Zhu, and Lu WANG
ARTICLE TYPE: Research Article

I am happy to tell you that your manuscript has been accepted for publication in Development, pending our standard ethics checks.

Reviewer 1

Advance summary and potential significance to field

The authors have adequately addressed my concerns in their revision of the manuscript, and I find it suitable for publication in Development.

Comments for the author

The authors have adequately addressed my concerns in their revision of the manuscript, and I find it suitable for publication in Development.

Reviewer 2

Advance summary and potential significance to field

The authors have addressed all of the questions I raised and I think it should be accepted now if Development has enough publication space.

Comments for the author

The authors have addressed all of the questions I raised and I think it should be accepted now if Development has enough publication space.

© 2022. Published by The Company of Biologists under the terms of the Creative Commons Attribution License (https://creativecommons.org/licenses/by/4.0/).
